# Sensory-Evoked Intrinsic Imaging Signals in the Olfactory Bulb Are Independent of Neurovascular Coupling

**DOI:** 10.1016/j.celrep.2015.06.016

**Published:** 2015-07-02

**Authors:** Roberto Vincis, Samuel Lagier, Dimitri Van De Ville, Ivan Rodriguez, Alan Carleton

**Affiliations:** 1 Department of Basic Neurosciences, School of Medicine, University of Geneva, 1211 Geneva, Switzerland; 2 Geneva Neuroscience Center, University of Geneva, 1211 Geneva, Switzerland; 3 Department of Radiology and Medical Informatics, University of Geneva, 1211 Geneva, Switzerland; 4 Institute of Bioengineering, Ecole Polytechnique Fédérale de Lausanne, 1015 Lausanne, Switzerland; 5 Department of Genetics and Evolution, University of Geneva, 1211 Geneva, Switzerland

## Abstract

Functional brain-imaging techniques used in humans and animals, such as functional MRI and intrinsic optical signal (IOS) imaging, are thought to largely rely on neurovascular coupling and hemodynamic responses. Here, taking advantage of the well-described micro-architecture of the mouse olfactory bulb, we dissected the nature of odor-evoked IOSs. Using in vivo pharmacology in transgenic mouse lines reporting activity in different cell types, we show that parenchymal IOSs are largely independent of neurotransmitter release and neurovascular coupling. Furthermore, our results suggest that odor-evoked parenchymal IOSs originate from changes in light scattering of olfactory sensory neuron axons, mostly due to water movement following action potential propagation. Our study sheds light on a direct correlate of neuronal activity, which may be used for large-scale functional brain imaging.

## Introduction

In the last decades, imaging techniques have allowed us to watch the brain at work with extraordinary details and have provided an in-depth understanding of how neural networks function. In humans, non-invasive imaging techniques do not directly measure electrical signals but rather measure correlates of neuronal activity. Functional MRI (fMRI) of blood-oxygen-level-dependent (BOLD) contrast relies on changes in blood oxygenation in active brain regions (Logothetis and Wandell, 2004; Logothetis et al., 2001). Intrinsic imaging, either called intrinsic optical signals (IOSs) imaging, near-infrared spectroscopy (NIRS), or 2D optical imaging spectroscopy (2D-OIS), is thought to reflect cerebral blood flow and oxygenation level changes (Grinvald et al., 1999; Martin et al., 2002; Murkin and Arango, 2009), whereas diffusion fMRI measures water diffusion (Le Bihan et al., 2006). Because all these measurements are indirect, it is crucial to understand their relation to neuronal activity to properly interpret functional brain-imaging data.

In animal models, IOSs have been used as a surrogate of BOLD-fMRI to study neurovascular coupling (Berwick et al., 2002; Cardoso et al., 2012; Niessing et al., 2005; Schummers et al., 2008; Sirotin and Das, 2009). Additionally, they have been extensively used for brain mapping in different species and several brain regions: visual, somatosensory, auditory, and gustatory cortices (Accolla et al., 2007; Accolla and Carleton, 2008; Frostig et al., 1990; Grinvald et al., 1986; Harrison et al., 1998), as well as the olfactory bulb (OB) (Abraham et al., 2004, 2014; Meister and Bonhoeffer, 2001; Rubin and Katz, 1999; Vincis et al., 2012). This technique reports changes in brain-tissue reflectance induced by neuronal activity (Grinvald et al., 1999). Such changes depend on incident light absorption by intrinsic chromophores and incident light scattering by the tissue refraction index inhomogeneities (Grinvald et al., 1999; Zepeda et al., 2004). At longer wavelengths (650–850 nm), variations in light scattering are thought to dominate IOS sources (Cohen et al., 1968; Frostig et al., 1990; Grinvald et al., 1999). At shorter wavelengths (450–650 nm), hemoglobin absorbance dominates, with variations in absorbance levels between oxy- and deoxyhemoglobin (Frostig et al., 1990). Both variations in blood flow and oxygenation can contribute to IOSs. It has therefore been proposed that, at shorter wavelengths (450–650 nm), IOSs originate from hemodynamics, following astrocyte-mediated neurovascular coupling (Gurden et al., 2006; Schummers et al., 2008).

In the present study, we unexpectedly found that stimulus-evoked parenchymal IOSs in the OB are independent of neurovascular coupling. We present evidence that parenchymal IOSs in the OB mainly come from the activity of olfactory sensory neuron (OSN) axons and are independent of neurotransmitter release. Our findings represent a significant step forward in understanding the origin of IOSs and provide crucial information about the different physiological correlates of neuronal activity that can be monitored by large-scale non-invasive functional imaging techniques.

## Results

### Parenchymal IOSs Are Independent of Hemodynamics in the OB

**Figure 1:**
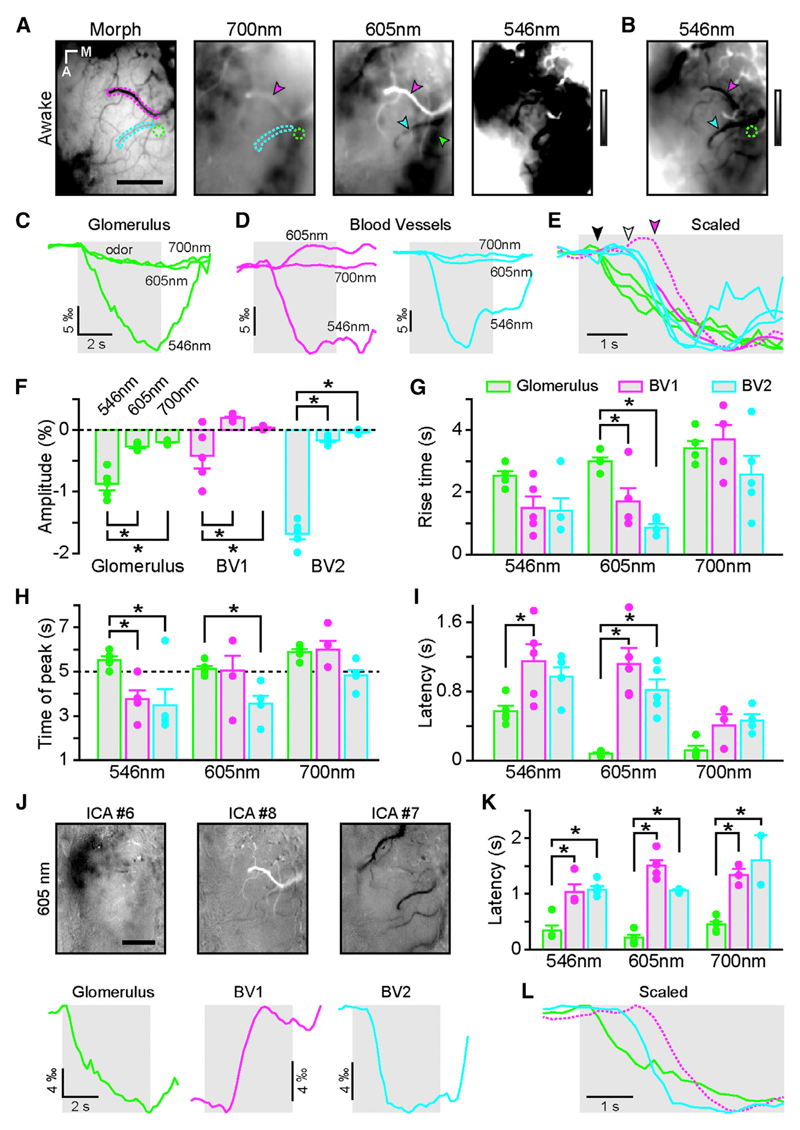
Odor-Evoked Glomerular and Vascular Intrinsic Optical Signals Have Different Kinetics in Awake Mice (A) Blood vessel pattern of the dorsal OB (Morph) and IOS glomerular map evoked by ethyl butyrate (5% in air) recorded at three different wavelengths in the same animal (gray look-up table [LUT]: −0.005 to 0.005 ΔR/R). Green and cyan/magenta dotted lines and arrowheads indicate regions of interest (ROIs) placed over an activated glomerulus and blood vessels, respectively. (B) Rightmost image shown in (A) (546 nm) after adjusting the look-up table (−0.02 to 0.02 ΔR/R). (C) IOS time course of the glomerulus marked in (A) and (B) (green ROI and arrow) at the different wavelengths. (D) IOS time course of the blood vessels marked in (A) and (B) (magenta and cyan ROIs and arrowheads) at the different wavelengths. Note the positive IOSs in the blood vessel marked in magenta at 605 nm. (E) IOS traces shown in (C) and (D) but scaled for comparison. The magenta dotted line has been flipped for better comparison. Note the delay from odor onset of the blood vessel response (white and magenta arrowheads) compared to glomerular ones (black arrowhead). (F) Average amplitude of IOSs recorded in glomeruli (green) and in blood vessels (magenta and cyan). Note the difference in amplitude between the two groups of blood vessels, particularly at 605 nm. (G) Average rise time measured between 20% and 80% of the response amplitude in glomeruli (green) and in blood vessels (magenta and cyan). Two-way ANOVA with repeated-measures F(2, 12) = 7.67; p = 0.007 for the ROI effect with Tukey’s correction for post hoc multiple comparison analysis. (H) Time of the peak of the IOS responses in glomeruli (green) and in blood vessels (magenta and cyan) relative to odor onset. The dashed line at 5 s represents the end of the odor application. Two-way ANOVA with repeated-measures F(2, 12) = 10.6; p = 0.002 for the ROI effect with Tukey’s correction for post hoc multiple comparison analysis. (I) Latency from odor onset of the IOS responses in glomeruli (green) and in blood vessels (magenta and cyan). Two-way ANOVA without repeated-measures F(2, 33) = 13.2; p < 10^−4^ for the ROI effect with Tukey’s correction for post hoc multiple comparison analysis. (J) Independent component analysis on the raw ΔR/R time series extract glomerular signal (ICA no. 6), positive signals in blood vessels (ICA no. 8), and negative signals in blood vessels (ICA no. 7) at 605 nm. The same recording presented in (A)–(E) was used here. The temporal profiles extracted by the ICA are presented at the bottom whereas Z score maps are presented above. Note that the antero-medial and the postero-lateral clusters of glomeruli are often separated by the ICA (compare A and J). (K) Latency from odor onset of the IOS responses in glomeruli (green) and in blood vessels (magenta and cyan) calculated from the ICA waveforms. Two-way ANOVA without repeated-measures F(2, 30) = 92.6; p < 10^−4^ for the ROI effect with Tukey’s correction for post hoc multiple comparison analysis. (L) ICA waveforms shown in (J) scaled for comparison. The magenta dotted line has been flipped for better comparison. Note the delay from odor onset of the blood vessel response (cyan and especially magenta) compared to glomerular ones. The scale bars represent 400 µm in (A) and (J). The light gray boxes represent odor application. * indicates corrected post hoc test p < 0.05. For clarity, significant comparisons are only indicated between wavelength (in F) or between ROI types (in G–K). Data are presented as mean ± SEM. See also Figure S1.

To study the origin of in vivo stimulus-evoked intrinsic signals, we first assessed whether odor-evoked IOSs were dependent on hemodynamic changes in awake head-restrained mice (Gschwend et al., 2012; Vincis et al., 2012). We recorded IOSs elicited by different odorant stimuli, varied the wavelength of the incident light, and quantified the amplitude and kinetics of activated regions of interest over time (Figures 1A–1I). At all wavelengths, we found discrete circular-shaped activated areas corresponding to OB glomeruli (Abraham et al., 2004; Bathellier et al., 2007, 2008; Belluscio et al., 2002; Meister and Bonhoeffer, 2001; Rubin and Katz, 1999; Uchida et al., 2000; Vincis et al., 2012) (Figures 1A and 1B). The amplitude of glomerular IOSs increased at shorter wavelengths, always staying negative (Figures 1C and 1F). In contrast, stimulus-evoked IOSs in blood vessels were barely detectable at longer wavelength and varied in sign at shorter wavelength (Figures 1D and 1F). Indeed, at 605 nm, some blood vessels showed an increase in reflectance (whitening in Figure 1A) whereas others showed a decrease in reflectance (darkening in Figure 1A). We split these two populations for further quantifications (Figure 1F). Almost all blood vessels displayed a decrease in reflectance at 546 nm. The blood vessels exhibiting negative IOSs at 605 nm had the largest (negative) IOSs at 546 nm. The shapes of the IOSs from glomeruli and blood vessels were strikingly different. Glomerular responses started shortly after stimulus onset and grew continuously until stimulus offset. In contrast, blood vessel responses were delayed and reached a peak before the stimulus offset (Figures 1G and 1I). Additionally, we observed a tendency for positive IOSs in blood vessels to arrive later than negative signals (Figures 1E, 1I, and S1). In order to avoid a potential bias originating from the manual selection of regions of interest, we also analyzed our data using independent component analysis (ICA). This unbiased approach led to the same results, separating blood vessels in two categories according to the sign of their response at 605 nm and revealing a delayed latency from stimulus onset in blood vessels relative to glomeruli (Figures 1J–1L). Altogether, these observations show that, in awake mice, the kinetics of glomerular IOSs is significantly different from the ones arising from blood vessels.

**Figure 2:**
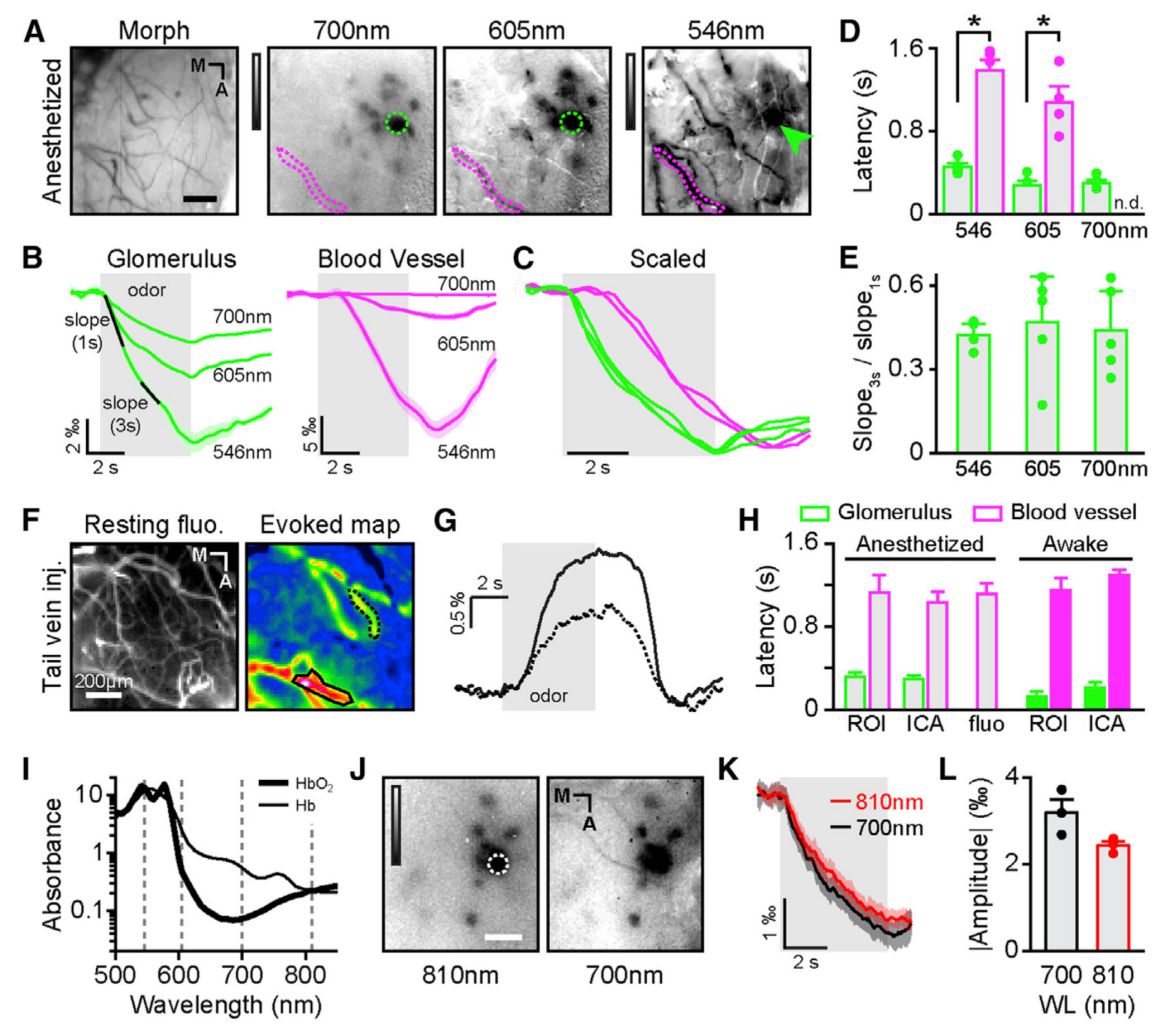
Parenchymal Intrinsic Optical Signals of Glomerular-Evoked Activity Do Not Depend on Hemodynamics (A) Blood vessel pattern of the dorsal OB (Morph) and IOS glomerular map evoked by methyl benzoate (5% in air) recorded at three different wavelengths in the same anesthetized animal (gray LUT: −0.0035 to 0.0015 ΔR/R for 700 and 605 nm and −0.01 to 0.006 ΔR/R for 546 nm). Green and magenta dotted lines indicate ROIs placed over an activated glomerulus and blood vessel, respectively. (B) IOS time course of the glomerulus and the blood vessel marked in (A) (green and magenta ROIs and arrow, respectively) at the different wavelengths. (C) IOS traces shown in (B) normalized for comparison. Note the delay from odor onset of the blood vessel traces (magenta) compared to glomerular ones (green). (D) Average response latencies computed for glomeruli (green) and blood vessels (magenta; Mann-Whitney *U* test; glomeruli versus blood vessels; n = 5 mice except for blood vessels at 605 nm, n = 4 mice; U(8) = −2.5, p = 0.008 and U(7) = 0, p = 0.016 at 546 and 605 nm, respectively). n.d., not determined. (E) Average slope ratios of the glomerular responses measured at different wavelengths (3^rd^ second over 1^st^ second response period; see B). One point represents one animal and is an average of many glomeruli (Friedman test; p = 0.52; F = 1.6). (F) (Left) resting fluorescence on the dorsal OB after fluorescein dextran injection in the tail vein. (Right) Average map of blood flow change evoked by ethyl butyrate is shown. The black outlined areas point out examples of odor-evoked activated ROIs. (G) Temporal profiles of the two ROIs marked in (F). (H) Comparison of IOSs and hemodynamic response latencies obtained in different conditions and with different techniques. The columns labeled ROI correspond to manually selected ROIs in anesthetized animals (gray bars; data from Figure 2D) and in awake mice (color-filled bars; data from Figure 1I). The columns labeled ICA correspond to the ICA analysis on data from anesthetized mice (gray bars) and in awake mice (color-filled bars; data from Figure 1K). The middle bar (fluo) corresponds to the data collected in anesthetized mice after a tail vein injection of fluorescein-dextran. There are no statistically significant differences between the latencies of IOSs measured in blood vessel in the five conditions (Kruskal-Wallis; K(5,27) = 3.28; p = 0.51). All glomeruli latencies are significantly smaller than any latency from blood vessels. ANOVA with Fisher LSD post hoc test F(1,36) = 178; p < 10^−4^. (I) Absorption spectra of oxyhemoglobin (HbO) and deoxyhemoglobin (Hb) (data from Takatani and Graham, 1979), plotted on a logarithmic axis. Black dotted line highlights wavelength at which IOS recording is performed. (J) IOS glomerular maps evoked by methyl benzoate (3.33% in air) recorded at two different wavelengths (810 and 700 nm; LUT: −0.0035 to 0.0015 ΔR/R). (K) Time course of the activation of the glomerulus marked in (J), imaged at 700 nm and 810 nm. (L) Average amplitude across mice of IOSs recorded at 700 nm (black) and 810 nm (WL, wavelength; red; paired t test; n = 3; t(2) = 3.03; p = 0.094). The scale bars represent 100 µm in (A) and (I) and 200 µm in (F). Data are presented as mean ± SEM. See also Figure S2.

In order to further pharmacologically dissect out the origin of IOSs in the OB, we planned to carry out experiments in anesthetized mice. Knowing that a number of anesthetics affect neurovascular coupling (Masamoto and Kanno, 2012; Nakao et al., 2001), we first checked that our conclusions from recordings in awake mice still held under anesthesia. The main effect of our anesthetics was to slow down the kinetics of vascular IOSs without affecting the kinetics of glomerular IOSs (Figures 2A–2E; amplitude awake = 1.95‰ ± 0.14, 2.69‰ ± 0.24‰, and 8.76‰ ± 1.01‰ at 700, 605, and 546 nm, respectively; amplitude anesthetized = 2.27‰ ± 0.3‰, 3.75‰ ± 0.79‰, and 6.28‰ ± 1.32‰ at 700, 605, and 546 nm, respectively; two-way repeated-measures ANOVA; F(1,9) = 0.17; p = 0.69; 20%–80% rise time awake versus anesthetized; two-way repeated-measures ANOVA; F(1,9) = 0.41; p = 0.54). Additionally, no significant increase in reflectance (i.e., whitening [i.e., positive IOSs]) was observed in blood vessels. Most importantly, our previous finding on latency discrepancy between vascular and glomerular IOSs still held (Figures 2B–2D). Indeed, glomerular odor-evoked IOSs had always shorter latency from stimulus onset (455 ± 31 ms at 546 nm and 278 ± 42 ms at 605 nm) than blood vessels IOSs (1,384 ± 108 ms at 546 nm and 1,077 ± 136 ms at 605 nm). These results were obtained when analyzing the time course of the local component of intrinsic signal, which is extracted by filtering out specific spatial frequencies from the raw data. However, similar results were found on the raw (unfiltered) and diffuse component of IOSs (Figure S2), therefore ruling out a potential bias introduced by the spatial filter.

A direct measurement of blood flow changes (by injection of fluorescein dextran in the bloodstream) gave us the same latency values, with hemodynamics lagging odor onset by more than 1 s (Figures 2F and 2G). This delay matched the values of vascular IOSs (Figure 2H). In our recording conditions, vascular IOSs are thus likely to reflect mostly changes in blood flow. With different recording techniques (fluorescence and IOS), different analyses (ROI-based and ICA), and different levels of arousal (awake and under anesthesia), glomerular IOSs consistently preceded blood vessel response by about 1 s (Figure 2H). This latency discrepancy led us to conclude that changes in blood flow do not contribute to the initial rise of intrinsic signals we recorded in activated glomeruli.

However, one has to note that the delay in vascular IOSs does not necessarily mean that vascular changes do not contribute at all to glomerular IOSs. Indeed, it is possible that a late component of glomerular IOSs stems from hemodynamics. Given the difference in wavelength dependency between IOSs originating from light scattering and from hemodynamics (Frostig et al., 1990), we compared the slope of the initial glomerular response to the slope of that response between 3 and 4 s after the odor onset (Figure 2B). This ratio was constant at the three wavelengths recorded (Figure 2E), therefore ruling out the contribution of a source of different origin than that of the initial response.

Oxyhemoglobin (HbO) (i.e., hemoglobin carrying oxygen molecules) has a different absorption spectrum than deoxyhemoglobin (Hb) (Figure 2I). It is thus possible that changes in the ratio of HbO to Hb contribute to changes in IOSs. In the first second of IOSs, the total amount of hemoglobin (HbT) being constant (see previous paragraphs), changes in HbO and Hb concentrations should be equal and opposite. As a consequence, signals recorded at isosbestic points (i.e., when Hb and HbO absorbances are equal) should be flat. Likewise, signals should be maximal when the difference in absorbance is the largest. In our recordings, we found the highest amplitude at 546 nm, close to an isosbestic point, and comparable amplitudes at 700 and 810 nm (Figures 2J–2L), two wavelengths at which the differences in absorbance are very different (Figure 2I).

As a conclusion, neither blood flow nor blood oxygenation changes can explain the first second of the glomerular intrinsic signals. Given the wavelength invariance of the response waveform, we conclude that vascular and parenchymal IOSs have different origins and need to be treated separately. In the OB, parenchymal IOSs do not result from hemodynamic features such as blood volume and oxygenation and thus must originate from variations in light scattering. In order to investigate which cellular components of the OB network give rise to light scattering changes, we pharmacologically dissected out the origin of in vivo stimulus-evoked IOSs.

### Parenchymal IOSs in the OB Originate from OSN Activity

**Figure 3:**
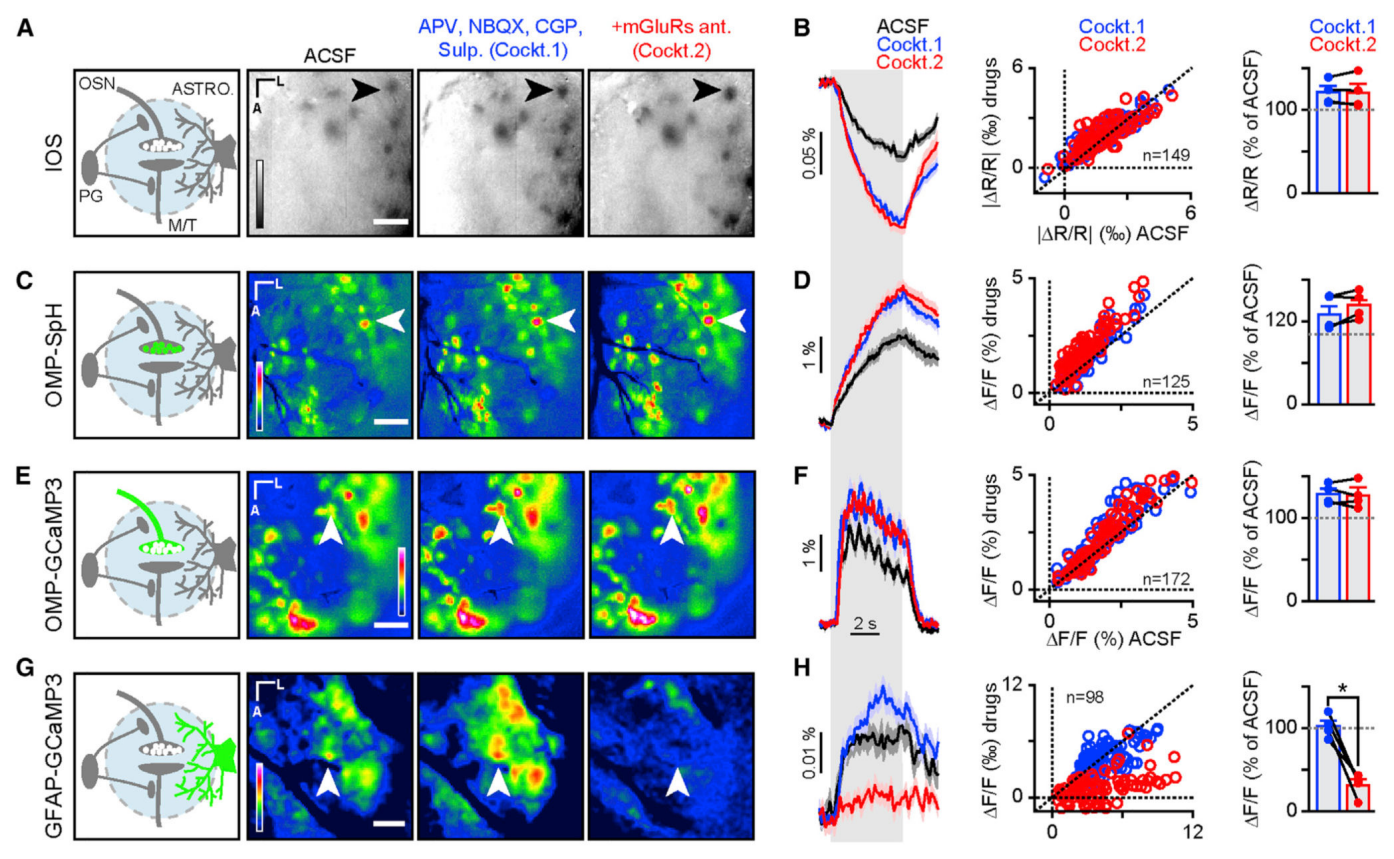
Parenchymal IOSs Are Independent of Post-synaptic Activity and Neurovascular Coupling (A) (Left panel) Schematic representation of OB glomerular circuitry. Right images show average maps of odor-evoked activity reported by IOSs in control condition (ACSF) and after topical application of a first drug mix (Cockt. 1) containing APV (1 mM), NBQX (0.1 mM), CGP (1 mM), and sulpiride (1 mM) and of a second drug mix (Cockt. 2) containing Cockt.1 with addition of mGluRs antagonist MCPG (100 µM), CPPG (10 µM), and LY 341495 (2 µM). The reflectance of IOSs is represented as ΔR/R (see Experimental Procedures). LUT: −0.01 to 0.006 ΔR/R. Black arrows point to an example of odor-evoked activated glomeruli. The scale bar represents 100 µm. (B) (Left panel) Traces showing the time course of odor-evoked activity from the glomerulus marked in (A) for three conditions (ACSF, black; Cockt.1, blue; and Cockt.2, red; mean ± SEM of eight trials for each condition). Light gray vertical bar represents odor stimulation. (Central panel) Average values of glomeruli amplitude response after Cockt.1 (blue circles) and Cockt.2 (red circles) application are shown, plotted against amplitude values in control condition (ACSF; Wilcoxon signed-rank test; Cockt.1 versus Cockt.2; p = 0.09; n = 149 glomeruli from four mice). (Right panel) Average values of glomeruli amplitude response after application of Cockt.1 (blue) and Cockt.2 (red) across mice are shown. Values are normalized relative to control condition (ACSF; paired t test t(3) = −0.032; p = 0.98; n = 4 bulbs from four mice). (C) Same drug conditions as in (A). (Left panel) The location of the fluorescent reporter is represented in green. (Right images) Average map of odor-evoked activity reported by OMP-SpH is shown. White arrows point to an example of odor-evoked activated glomeruli. The fluorescence is represented as ΔF/F (see Experimental Procedures). LUT: −0.015 to 0.05 ΔF/F. The scale bar represents 100 µm. (D) Same as in (B). (Left panel) Traces show the time course of odor-evoked activity from the glomerulus marked in (C) for three conditions (mean ± SEM of eight trials for each condition). (Central panel) Wilcoxon signed-rank test is shown; Cockt.1 versus Cockt.2; p = 1e−12; n = 125 glomeruli from four mice. (Right panel) Paired t test is shown; t(3) = −1.1; p = 0.35; n = 4 bulbs from four mice. (E) Same drug conditions as in (A). (Left panel) The location of the fluorescent reporter is represented in green. (Right images) Average map of odor-evoked activity reported by OMP-GCaMP3 is shown. White arrows point to an example of odor-evoked activated glomeruli. The fluorescence is represented as ΔF/F (see Experimental Procedures). LUT: −0.015 to 0.055 ΔF/F. The scale bar represents 100 µm. (F) Same as in (B). (Left panel) Traces show the time course of odor-evoked activity from the glomerulus marked in (E) for three conditions (mean ± SEM of eight trials for each condition). (Central panel) Wilcoxon signed-rank test is shown; Cockt.1 versus Cockt.2; p = 4.0e−1; n = 172 glomeruli from four mice. (Right panel) Paired t test is shown; t(3) = −0.033; p = 0.76; n = 4 bulbs from four mice. (G) Same drug conditions as in (A). (Left panel) The location of the fluorescent reporter is represented in green. (Right images) Average map of odor-evoked activity reported by GFAP-GCaMP3 is shown. White arrows point to an example of odor-evoked activated glomeruli. The fluorescence is represented as ΔF/F (see Experimental Procedures). LUT: −0.0005 to 0.007 ΔF/F. The scale bar represents 100 µm. (H) Same as in (B). (Left panel) Traces show the time course of odor-evoked activity from the glomerulus marked in (G) for three conditions (mean ± SEM of eight trials for each condition). (Central panel) Wilcoxon signed-rank test is shown; Cockt.1 versus Cockt.2; p = 1.6e−17; n = 98 glomeruli from four mice. (Right panel) Paired t test is shown; t(3) = 7.0; p = 0.0059; n = 4 bulbs from four mice. Data are presented as mean ± SEM. See also Figures S3–S5.

OSNs’ axonal projections coalesce in the OB glomeruli, where they form axo-dendritic glutamatergic synapses with the dendritic tufts of both output neurons (mitral and tufted [M/T] cells) and a heterogeneous population of interneurons called periglomerular (PG) cells (Berkowicz et al., 1994; Ennis et al., 1996) (Figure 3A). Glutamate released from OSNs activates post-synaptic ionotropic AMPA and NMDA receptors located on M/T and PG cells (Aroniadou-Anderjaska et al., 1997; Ennis et al., 1996). The activation of PG cells, in turn, triggers the release of dopamine and GABA, which inhibits OSNs via presynaptic metabotropic D_2_ and GABA_B_ receptors (Ennis et al., 1996; Hsia et al., 1999; McGann et al., 2005; Petzold et al., 2008). All synaptic contacts between these cell types lie within 200 µm of OB’s surface, thus rendering them accessible to in vivo pharmacological manipulation. In order to monitor the activity of the cellular partners of the OB circuitry, we used mouse lines expressing the Ca^2+^-sensitive fluorescent protein GCaMP3 in PCDH21-expressing cells (i.e., M/T cells), in OMP-expressing cells (i.e., OSNs), and in GFAP-expressing cells (i.e., astrocytes). We additionally used a mouse line reporting synaptic vesicle fusion in OSNs (OMP-synaptopHluorin [OMP-SpH]; Bozza et al., 2004). These mouse lines, reporting activity from different synaptic partners, allowed us to carefully control for the specificity of drug effects on odor-evoked IOSs.

We first made sure we could block OSN to M/T cell glutamatergic synaptic transmission in vivo with AMPA and NMDA receptor antagonists (Figures S3A–S3E). Second, we verified in OMP-SpH mice that pharmacological modulation of GABA_B_ (Figures S3F–S3J) and D_2_ (Figures S3K–S3O) receptors altered release from OSN terminals. After validation of our pharmacological approach, we tested whether IOSs originated from post-synaptic neurons. The application of APV, NBQX, sulpiride, and CGP35348 (NMDA, AMPA, D_2_, and GABA_B_ receptor antagonists, respectively; hereinafter referred to as Cockt.1) did not decrease but rather increased IOS amplitude (Figures 3A and 3B). Similar results were obtained on different IOS components at different wavelengths (Figure S4). An increase in signal amplitude was similarly observed in OMP-SpH and OMP-GCaMP3 mice, which most likely reflects the removal of endogenous presynaptic inhibition of OSNs by PG cells through D_2_ and GABA_B_ receptors (Figures 3A–3F). Our data thus suggest that IOSs do not depend on post-synaptic neuronal activity.

**Figure 4:**
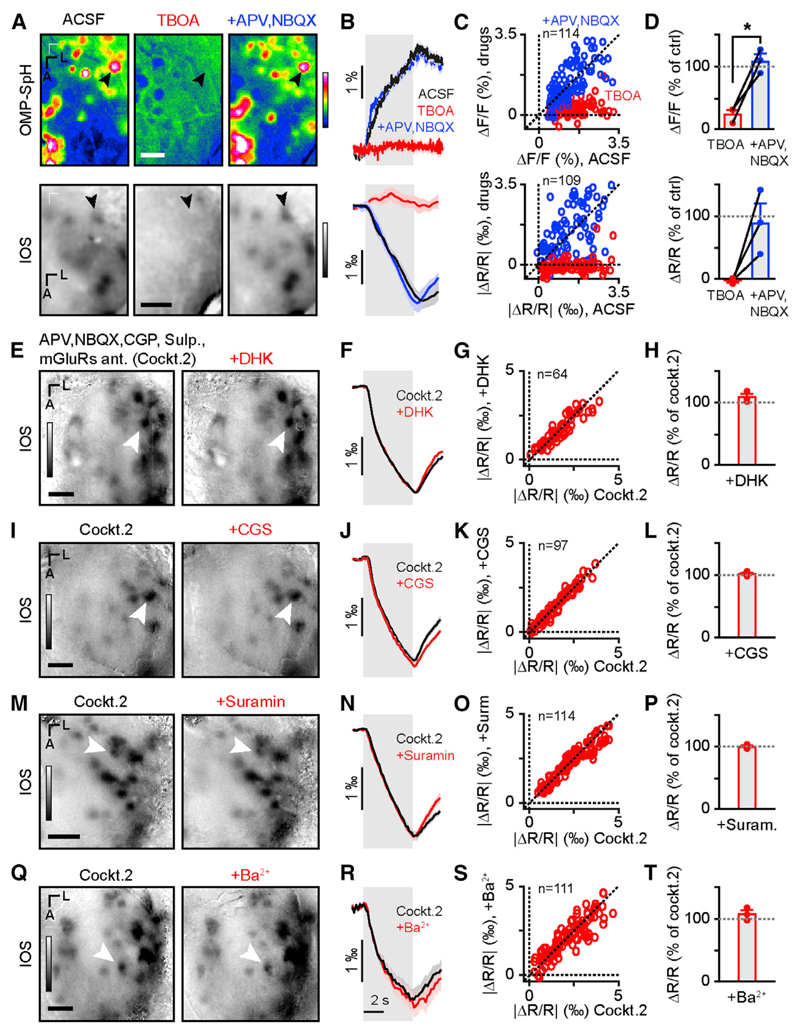
Parenchymal IOSs Do Not Depend on Astrocytic Activity (A) Average map of odor-evoked glomerular activity measured by fluorescence in OMP-SpH mice (top row) and IOSs in WT mice (bottom row) in control conditions (ACSF) in the presence of 5 mM TBOA and in the presence of TBOA, NBQX, and APV (5 mM, 0.1 mM, and 1 mM, respectively). LUT: −0.01 to 0.03 ΔF/F and −0.02 to 0.03 ΔR/R for the fluorescence and IOSs, respectively. (B) Time course of odor-evoked activity from the glomeruli marked in (A) for three pharmacological conditions (mean ± SEM of eight trials). (C) Individual glomerular response amplitude during drug applications plotted against amplitude values in ACSF condition in fluorescence (top; Wilcoxon signed-rank test; n = 114 glomeruli from three mice, Z(114) = −9.7, p = 1.9e−20 and Z(114) = −0.94, p = 0.35 for ACSF versus TBOA and ACSF versus +APV, NBQX, respectively) and in IOSs (bottom; Wilcoxon signed-rank test; n = 109 glomeruli from three mice; Z(109) = −9.1; p = 1.3e−19 and Z(109) = −0.016, p = 0.99 for ACSF versus TBOA and ACSF versus +APV, NBQX, respectively). (D) Average amplitude across mice in fluorescence (top; paired t test; n = 3 mice; TBOA versus +NBQX, APV; t(2) = −6.1; p = 0.025) and in IOSs. (E) Average map of odor-evoked IOSs in control condition (Cockt.2) and in presence of DHK (1 mM). LUT: −0.002 to 0.003 ΔR/R. (F) Time courses of odor-evoked activity from the glomerulus marked in (E) (mean ± SEM of eight trials). (G) Individual glomerular IOS amplitude in presence of DHK plotted against amplitude values in Cockt.2 condition (Wilcoxon signed-rank test; n = 64 glomeruli from three mice; Z(64) = −0.12; p = 0.90). (H) Average IOS amplitude across mice (n = 3 bulbs from three mice; 108% ± 9% of Cockt.2). (I) Average map of odor-evoked IOSs in control condition (Cockt.2) and in presence of CGS (0.5 mM). LUT: −0.002 to 0.003 ΔR/R. (J) Time courses of odor-evoked activity from the glomerulus marked in (I) (mean ± SEM of eight trials). (K) Individual glomerular IOS amplitude in presence of CGS plotted against amplitude values in Cockt.2 condition (Wilcoxon signed-rank test; n = 97 glomeruli from three mice; Z(97) = −1.5; p = 0.14). (L) Average amplitude across mice expressed as % of control condition (n = 3 bulbs from three mice; 100.7% ± 2% of Cockt.2). (M) Average map of odor-evoked IOSs in control condition (Cockt.2; left image) and in presence of suramin (2 mM; right). LUT: −0.002 to 0.003 ΔR/R. (N) Time courses of odor-evoked activity from the glomerulus marked in (M) (mean ± SEM of eight trials). (O) Individual glomerular IOS amplitude in presence of suramin plotted against amplitude values in Cockt.2 condition (Wilcoxon signed-rank test; n = 114 glomeruli from three mice; Z(114) = −1.9; p = 0.059). (P) Average IOS amplitude across mice (n = 3 bulbs from three mice; 97% ± 2% of Cockt.2). (Q) Average map of odor-evoked IOSs in control condition (Cockt.2; left) and in presence of Ba^2+^ (1 mM; right). LUT: −0.002 to 0.003 ΔR/R. (R) Time courses of odor-evoked activity from the glomerulus marked in (Q) (mean ± SEM of eight trials). (S) Individual glomerular IOS amplitude in presence of Ba^2+^ plotted against amplitude values in Cockt.2 condition (Wilcoxon signed-rank test; n = 111 glomeruli from three mice; Z(111) = −0.50; p = 0.62). (T) Average IOS amplitude across mice (n = 3 bulbs from three mice; 105% ± 6% of Cockt.2). Light gray box in (B), (F), (J), (N), and (R) represents odor presentation. The scale bars represent 250 µm. See also Figure S5.

Astrocytes are a key element of neurovascular coupling and have previously been reported to generate IOSs in the OB (Gurden et al., 2006). They can sense glutamate release through metabotropic receptors (mGluRs). We found that applying MCPG, CPPG, and LY mGluR antagonists (group I and II, group III, and group II receptor antagonists, respectively; the cocktail hereinafter referred to as Cockt.2 contains GluRs, mGluRs, D_2_R, and GABA_B_R antagonists) did indeed decrease astrocyte Ca^2+^ signals (Figures 3G and 3H). In contrast, blocking mGluRs had no effect on IOSs, OSN glutamate release, and OSN Ca^2+^ signals (Figures 3A–3F). Astrocytes also sense glutamate release through excitatory amino acid transporters (EAAT) (Attwell et al., 2010; Bernardinelli and Chatton, 2008; Danbolt, 2001). The use of the general EAAT blocker DL-TBOA alone proved to be unsuitable for our experiments given its effect on glutamate release from OSNs (Figures 4A–4D). Indeed, application of 5 mM DL-TBOA almost completely abolished vesicle fusion in OSNs in OMP-SpH mice. Moreover, this effect was reverted by the addition of ionotropic glutamate receptors (NBQX and APV; Figures 4A– 4D). Application of dihydrokainic acid (DHK), an astrocyte-specific EAAT blocker, also had no effect on IOSs (Figures 4E–4H). ATP released by OSNs (Thyssen et al., 2010) can be sensed, along with its metabolites (ADP and adenosine) by astrocytes (Doengi et al., 2008). The application of CGS and suramin (broad P1 and P2 receptor antagonists, respectively) did not affect IOSs (Figures 4I–4P). Astrocytes are also sensitive to extracellular K^+^ concentration. They play an important role in K^+^ siphoning via inward-rectifier K^+^ (K_ir_) channels, which is key in preventing epileptic activity (Attwell et al., 2010). Application of 1 mM Ba^2+^, a broad antagonist of K_ir_ channels, did not affect IOSs (Figures 4Q–4T). The same results were observed when analyzing the raw images and the diffuse component of IOSs (Figure S5). Likewise, DHK, suramin, CGS, Ba^2+^, and the mGluR antagonists had very little effect on the resting fluorescence (Figure S5).

Altogether, these results allow us to rule out the contribution of post-synaptic neurons and astrocytes to parenchymal IOSs measured in the OB. We can thus conclude that parenchymal IOSs in the OB do not reflect post-synaptic neuron activity nor do they reflect neurovascular coupling through astrocytic activity. Odor-evoked intrinsic signals in the OB rather reflect the activity of OSNs.

### Parenchymal IOSs Are Independent of Neurotransmitter Release from OSNs

**Figure 5:**
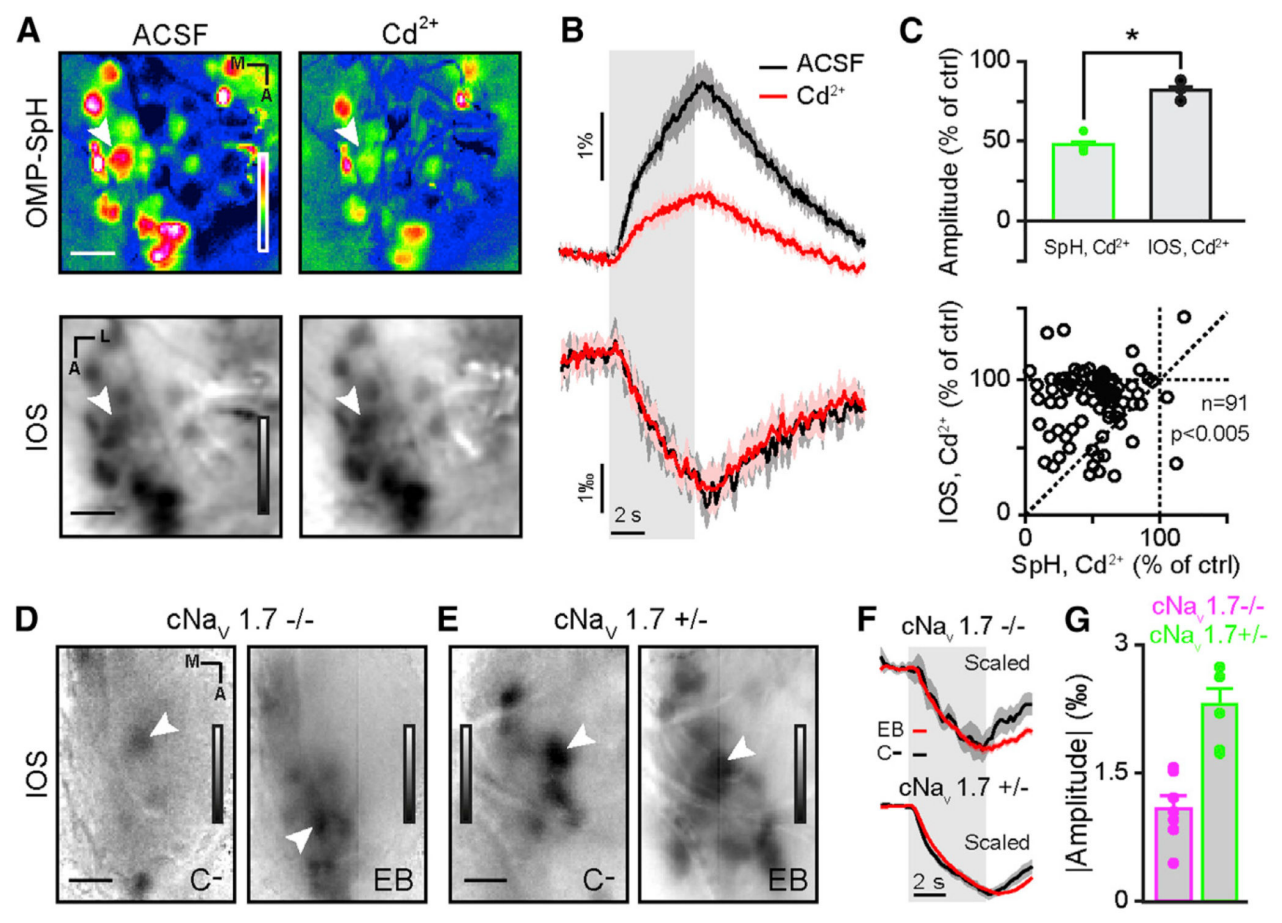
Parenchymal IOSs Are Independent of Neurotransmitter Release from OSNs (A) Average fluorescence (top) and IOS (bottom) olfactory maps evoked by ethyl butyrate (5% in air) in the same OMP-SpH animal. Images are recorded in ACSF (left) and in presence of Cd^2+^ (2 mM; right). LUT: SpH, −0.01 to 0.03 ΔF/F and IOS, −0.0045 to 0.0025 ΔR/R. (B) Time courses of SpH (top) and IOS (bottom) odor-evoked activity from the glomerulus marked in (A), in control condition (ACSF; black) and in Cd^2+^ (red). The area around the curve represents ± SEM. (C) (Top) Average SpH (green) and IOS (black) amplitude across mice in presence of Cd^2+^ (n = 3 bulbs from three mice; paired t test; t(2) = 5.3; p = 0.034). (Bottom) Individual glomeruli IOS activity (ordinate) as a function of their SpH activity (abscissa) in presence of Cd^2+^ (n = 91 glomeruli from three mice; Wilcoxon sign rank test; Z(91) = −6.5; p = 7e−11). (D and E) IOS olfactory map evoked by carvone-(left) and ethyl butyrate (right; 20% and 2% in air, respectively) in a cNa_v_1.7^−/−^ (D) and a cNa_v_1.7^+/−^ mouse (E). LUT: −0.0009 to 0.0003 (D; left image), −0.002 to 0.001 (E; right image) and −0.004 to 0.002 (D; left image), −0.006 to 0.002 (E; right image) ΔR/R. (F) Normalized traces of the activity of the glomeruli marked in (A) and (B) in a cNa_v_1.7^−/−^ mouse (top) and cNa_v_1.7^+/−^ mouse (bottom) evoked by ethyl butyrate (EB) (red) and carvone- (C-) (black). (G) Averaged odor-evoked IOS amplitudes recorded in cNa_v_1.7^−/−^ (magenta) and cNa_v_1.7^+/−^ mice (green; n = 6 and 7 bulbs from four and five mice for cNa_v_1.7^+/−^ and cNa_v_1.7^+/+^, respectively). The scale bars represent 200 µm in (A) and 250 µm in (D) and (E). Data are presented as mean ± SEM.

In order to further confirm that IOS origin is presynaptic, we used two approaches to block vesicular release from OSNs. Indeed, OSNs might release some unknown and/or unconventional transmitter (e.g., neuropeptides) along with glutamate. The receptor of such transmitter would have been missed in our initial pharmacological dissection. We first measured SpH and IOS odor-evoked activity in the same OMP-SpH animals before and after blocking voltage-gated Ca^2+^ (Ca_v_) channels with 2 mM CD^2+^. The application of this broad Ca_v_ antagonist reduced significantly vesicle fusion but had a minimal effect on intrinsic signals (Figures 5A–5C). These results suggest that neurotransmitter release and vesicle fusion are not major contributors to IOSs. In order to further ascertain that conclusion, we used a conditional null mouse lacking the voltage-gated Na^+^ channel Na_v_1.7 in OMP-expressing cells (i.e., OSNs; see Experimental Procedures; mice referred to as cNa_v_1.7^−/−^; Weiss et al., 2011). This Na^+^ channel subunit has been found to be the only one expressed in glomeruli and OSN axon terminals (Weiss et al., 2011). Its removal renders mice fully anosmic while preserving OSNs function, including action potential generation (Weiss et al., 2011). In this mouse line, the average amplitude of the signals was smaller than in heterozygous controls, but we could still record odor-specific intrinsic signals (Figures 5D–5G).

Altogether, these data suggest that odor-evoked parenchymal IOSs in the OB do not depend on the release of neurotransmitter or other messenger molecule by vesicle fusion from OSNs. Parenchymal IOSs are thus purely presynaptic and reflect changes in light-scattering properties of OSN axons in the OB.

### Altering Water Diffusion Disrupts IOSs without Impairing OSN Activity

**Figure 6:**
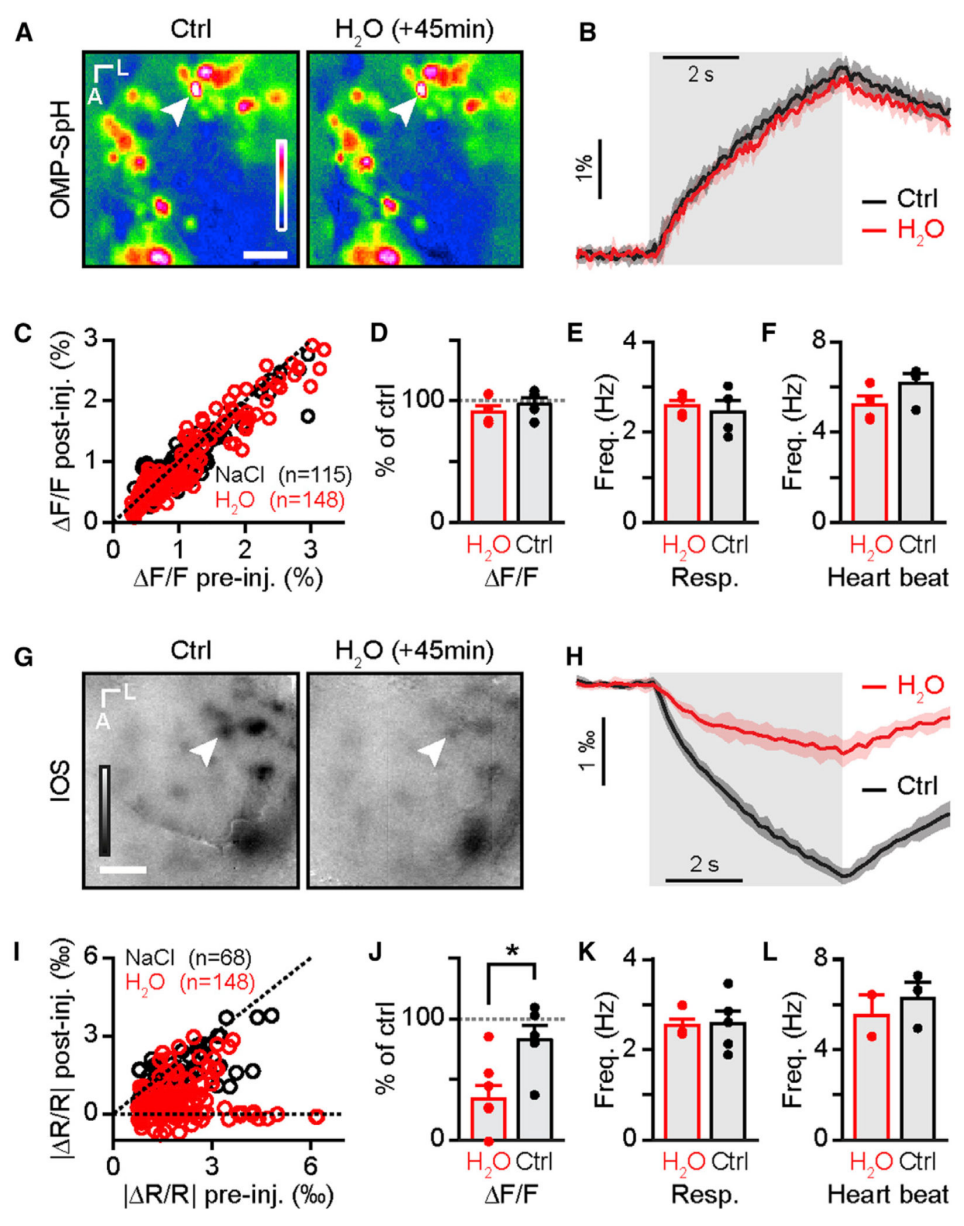
Parenchymal IOSs Are Sensitive to Extracellular Osmolarity (A) Odor-evoked olfactory maps recorded in an OMP-SpH mouse before (Ctrl; left) and 45 min after water intoxication (H_2_O; right). LUT: −0.01 to 0.03. (B) Time course of the activity of the glomerulus marked in (A) before (Ctrl; black) and 45 min after water intoxication (H_2_O; red). (C) Amplitude of individual glomeruli signals after injection (ordinate) as a function of the amplitude before injection (abscissa) of saline (NaCl; black) and water (H_2_O; red; n = 148 glomeruli from four mice for H_2_O condition and n = 115 glomeruli from four mice for NaCl condition; Mann-Whitney *U* test; U(261) = 8276; p = 0.70). (D) Averaged SpH amplitude across mice after saline (NaCl; black) and water (H_2_O; red; n = 4 mice from four mice for both water and saline injection; unpaired t test; *t*(6) = −0.75; p = 0.48). (E and F) Averaged breathing frequency (E) and heart beat (F) across mice after saline (NaCl; black) and water (H_2_O; red; n = 4 bulbs from four mice for water and saline injection, respectively; unpaired t test; t(6) = 0.87, p = 0.42 and t(6) = −2.9, p = 0.026 for resp. and heartbeat, respectively). (G) Odor-evoked IOS maps recorded before (Ctrl; left) and 45 min after water intoxication (H_2_O; right). LUT: −0.0025 to 0.0015. (H) Time course of the activity of the glomerulus marked in (G) before (Ctrl; black) and 45 min after water intoxication (H_2_O; red). (I) Amplitude of individual glomeruli signals after injection (ordinate) as a function of the amplitude before injection (abscissa) of saline (NaCl; black) and water (H_2_O; red; n = 148 glomeruli from six mice for H_2_O condition and n = 68 glomeruli from five mice for NaCl condition; Mann-Whitney *U* test; U(214) = 1,931; p = 3.6e−13). (J) Averaged IOS amplitude across mice after saline (NaCl; black) and water (H_2_O; red; n = 6 and 5 bulbs from six and five mice for H_2_O and NaCl condition, respectively; unpaired t test; t(9) = −2.8; p = 0.021). (K and L) Averaged breathing frequency (K; n = 4 and 5 bulbs from four and five mice for H_2_O and NaCl condition, respectively; unpaired t test; t(7) = −0.8957; p = 0.4002) and averaged heart beat (L; n = 2 and 3 bulbs from two and three mice for H_2_O and NaCl condition, respectively; unpaired t test; t(3) = −5.3467; p = 0.0128) after saline (NaCl; black) and water injection (H_2_O; red). The scale bars represent 250 µm in (A) and 100 µm in (G). Data are presented as mean ± SEM.

What could be the nature of changes in OSN axons leading to intrinsic signals? Activity-induced axonal swelling has been described in vitro (Fields and Ni, 2010; Tasaki and Byrne, 1983, 1988) and gives rise to measureable optical signals in vitro (Cohen et al., 1968; Tasaki et al., 1968). These optical changes reflect water and ion movements across the axonal membrane (Cohen, 1973). We hypothesized that in vivo functional IOSs are likewise caused by activity-induced cell swelling. An edema is a pathological disruption of the extracellular medium that results from altered diffusion of water and solutes. We used a standard model for general brain edema-water intoxication (i.e., systemic injection of distilled water; Manley et al., 2000) to assess the influence of osmolarity changes in the extracellular space on IOSs. We controlled for the effect of alterations in overall volume changes by injecting an isotonic solution (NaCl 0.9%; 310 mosm/l). We made sure that water intoxication had no effect on vesicle fusion in OSNs (Figures 6A–6D). Water intoxication can be lethal (Manley et al., 2000); we therefore controlled for vital parameters throughout our experiments and kept only data where the breathing and heartbeat of the animals were stable or slightly increasing (Figures 6E and 6F). In agreement with our hypothesis, we found that water intoxication disrupted IOSs (Figures 6G–6L). IOSs, as opposed to neurotransmitter release, are sensitive to the osmolarity of the extracellular space.

In conclusion, odor-evoked parenchymal IOSs are most likely generated by morphological changes caused by activity-induced solute and water movements across the membrane of OSN axons in the OB.

## Discussion

Our study assesses the nature of in vivo IOSs in the mouse OB. With a wide and complementary set of approaches, we show that odor-evoked IOSs recorded in glomeruli are unrelated to hemodynamics (Figures 1 and 2) and are independent of neurovascular coupling (Figures 3 and 4). Parenchymal IOSs are independent of neurotransmitter release and vesicle fusion (Figure 5) and depend on solute and water movements in OSN axons (Figure 6), which deliver peripheral olfactory inputs to the brain. Thus, parenchymal IOSs represent a different physiological correlate of neuronal activity than previously thought.

### Importance of the Brain Architecture for Light-Scattering Measurements

Intrinsic optical imaging has been used in the rodent olfactory system for more than a decade and has been shown to give functionally relevant “glomerular odor maps” (Belluscio et al., 2002; Rubin and Katz, 1999; Vincis et al., 2012). Indeed, glomeruli are the entry point of olfactory sensory information to the brain. They are morphologically and functionally well-defined structures where axons of OSNs expressing the same odorant receptor coalesce and form synapses on different OB neurons. Glomeruli are close to the pia, rendering them optically and pharmacologically accessible.

Our pharmacological approach aimed at exhaustively blocking channels, receptors, and transporters sensing OSN activity. None of the pharmacological manipulations significantly altered IOSs without affecting OSN activity (Figures 3, 4, and S3–S5). Another approach we took consisted in altering transmitter release from OSNs rather than blocking potential targets (Figure 5). The results of these experiments are consistent with a presynaptic origin of odor-evoked parenchymal IOSs. The addition of CD^2+^ did indeed reduce vesicle fusion with little effect on IOSs. Likewise, we could measure odor-evoked signals in mice lacking Na_v_ channels in OSN terminals. Though we cannot completely rule out a small contribution of other cell types, like astrocytes and bulbar neurons, our data suggest that most of odor-evoked parenchymal intrinsic signals we recorded arise from OSN axons.

One explanation for the predominant contribution of OSN axons to parenchymal IOSs is their massive convergence. It is estimated that 11,000 OSNs project in a given glomerulus in rodents (Shepherd et al., 2004). Moreover, OSNs that send their axons to a given glomerulus express the same odorant receptor (Ressler et al., 1994; Vassar et al., 1994), and their activity tends to be timed to the breathing cycle (Verhagen et al., 2007). They are thus conveying the same information synchronously in a spatially restricted location (Bozza et al., 2004; ~100 µm for the mouse OB glomerulus). In addition, the glomerular layer of the OB appears to be the brain region with the lowest extracellular space fraction (Zhang and Verkman, 2010) and consequently the highest density of neuropil. These features concur to maximize the contribution of OSN axons to IOS imaging in vivo. It is likely that the relative contribution of cellular elements to parenchymal IOSs may vary between brain regions, depending on their micro-architecture.

### IOSs and Hemodynamics

It is well known that neuronal activity precedes hemodynamic changes (Logothetis and Wandell, 2004). As early as 1890 was the correlation made between blood flow changes and brain activity (Roy and Sherrington, 1890), and changes in the level of blood oxygenation are at the basis of the development of BOLD fMRI in the 1990s (Ogawa et al., 1990). Vanzetta and Grinvald (1999) measured directly blood oxygenation levels and showed an initial increase in Hb after sensory stimulation followed by blood flow increase. Without the need for heavy equipment or molecular reporter, intrinsic imaging in the cortex reveals the same dynamics (Frostig et al., 1990; Grinvald et al., 1999), and extensive modeling has been developed to understand fine evolution of Hb, HbO, and HbT from intrinsic signals (Mayhew et al., 1999). In our experiments, we do observe changes in blood flow lagging glomerular IOSs by 1 s (Figures 1, 2, S1, and S2). One could argue that blood vessels distant from an activated region could respond later or that larger blood vessels have a response lagging the response in small capillaries. However, a similar time lag has been reported for capillaries located within activated glomeruli (Chaigneau et al., 2007; Lecoq et al., 2009, 2011; Otsu et al., 2015; Petzold et al., 2008). We can thus safely rule out the contribution of blood flow changes at least within the first second following stimulus onset. We did not observe changes in rise time as a function of wavelength; neither did we observe multiple components in the rising phase of our intrinsic signals, ruling out a major contribution of both blood oxygenation and flow changes throughout the entire response-time course.

Altogether, our data strongly argue against a significant impact of hemodynamics on parenchymal IOSs in the OB. Although the contribution of hemodynamics to IOSs may vary between brain areas, our data highlight the contribution of activity-dependent light-scattering signals, even at wavelengths usually considered reporting only hemodynamics. The interpretation of IOSs collected at such wavelengths might then not be straightforward and may explain some controversial IOS-based results failing to link local neuronal activity and hemodynamics (Handwerker and Bandettini, 2011; Kleinschmidt and Müller, 2010; Sirotin and Das, 2009).

### Astrocytes and Neurovascular Coupling

Astrocytes are an indispensable element of neurovascular coupling. Extensive work has been done on the topic, including in the OB, revealing the crucial role of astrocytes in K^+^ clearance (also called siphoning), glutamate reuptake, and vasodilation to shuttle nutrients to and metabolites from active brain regions (Attwell et al., 2010). A good part, if not all of these aspects rely on intracellular Ca^2+^ signaling (Carmignoto and Gómez-Gonzalo, 2010). In our experiments, neither K^+^ siphoning (blocked by Ba^2+^) nor glutamate reuptake (blocked by DHK) affected glomerular intrinsic signals, and the block of metabotropic glutamate receptors almost completely abolished odor-induced Ca^2+^ transients in astrocytes (Figures 3 and 4). One published work concluded from the disruption of odor-evoked IOSs by TBOA that these signals were arising from astrocyte swelling (Gurden et al., 2006). However, the use of this drug at the same concentration proves to block almost completely the release of glutamate by OSNs (Figures 4A–4C), rendering this experiment inconclusive. A further confirmation of the absence of implication of glutamate transporters in IOS generation came from the unexpected reversal of the TBOA effect on OSN activity by the addition of APV and NBQX (ionotropic glutamate receptor antagonists). Indeed, in the presence of TBOA, APV, and NBQX, conditions in which glutamate transporters are blocked (as well as ionotropic glutamate receptors), both SpH signals and IOSs are clearly visible (Figure 4C). A proper understanding of this surprising finding would require more investigation, but it reveals a misinterpretation of the data presented in Gurden et al. (2006).

### Morphological Correlates of Neuronal Activity and Relevance for Functional Brain Imaging

A number of studies have described shrinkage of the extracellular space induced by neuronal activity (Jarvis et al., 1999; Syková et al., 2003). However, none of these experiments were performed in vivo, and the conclusions diverge on the cellular basis of this phenomenon. In vitro, axons have been shown to swell (Cohen et al., 1968; Fields and Ni, 2010), as well as neuron cell bodies and astrocytes (Syková et al., 2003). These morphological changes are a consequence of the massive ion exchanges across neuronal and astrocytic membranes. The correlation between extracellular space shrinkage and IOSs has been studied in vitro and revealed that activity-dependent light scattering changes can indeed be optically recorded (Jarvis et al., 1999; Syková et al., 2003). However, the precise contribution of extracellular space shrinkage, fine morphological changes, protein packing, or cleavage is a matter of debate (Jarvis et al., 1999; Syková et al., 2003). In the present study, we did not attempt to measure morphological changes of neuronal and astrocytic processes directly. However, we manipulated the extracellular space and reproduced a pathological condition (edema) known to alter water diffusion (Manley et al., 2000; Schaefer et al., 2000). In this experiment, we saw a near extinction of IOSs with intact neurotransmitter release (Figure 6). Solutes and water movements across membranes are associated with cell swelling. It is thus likely that the observed morphological changes in OSNs generating odor-evoked IOSs are coming from the activity-induced swelling of their axons.

The change in optical properties measured with IOS imaging is an interesting correlate of neuronal activity and can be used in humans. Activity-induced changes in water diffusion have been measured with MRI in the human visual cortex (Le Bihan et al., 2006), and their correlation with neuronal activity was confirmed in the rat somatosensory cortex (Tsurugizawa et al., 2013). We hypothesize here that the signals measured with diffusion fMRI are of similar origin than the IOSs measured in our experiments. These two techniques can be used in humans and present the advantage to have faster kinetics than BOLD signals and to be less sensitive to anesthetics and drugs affecting the circulatory system. Additionally, signals from diffusion fMRI and IOSs are more spatially restricted to the activated neuronal networks than signals from BOLD fMRI and are therefore more accurate to map neuronal activity.

## Experimental Procedures

### Animals

All experiments were performed on 12- to 20-week-old male and female mice. We used C57BL/6J (Charles River France; for IOS imaging) and hemizygous *Omp^tm2^*^(*SpH*)^^*Mom*^ (Bozza et al., 2004; OMP-SpH; Jackson Laboratory; JAX 004946), and for calcium imaging experiments, we crossed mice conditionally expressing the calcium indicator GCaMP3 (Gt(ROSA)26Sortm38(CAG-GCaMP3)Hze/J; Zariwala et al., 2012; JAX 014538) with different cell-type-specific Cre-expressing lines. For OSNs, astrocytes, and M/T cells, we used *Omp^tm4^*^(*cre*)^^*Mom*^ (Li et al., 2004; OMP-Cre; JAX 006668; always kept hemizygous), B6.Cg-Tg505Fmv/J (GFAP-creERT; JAX 012849), and Tg(Pcdh21-creERT)CYoko (Yonekura and Yokoi, 2008; kind gift of Dr. Mineto Yokoi) mice, respectively. The creERT recombinase was activated by three intraperitoneal injections (one injection per day for 3 consecutive days, around postnatal day 21) of tamoxifen (100 µl; from a stock solution of 10 mg/ml tamoxifen in Corn Oil; Sigma-Aldrich). Finally, floxed Na_v_1.7 (Nassar et al., 2004) were crossed with OMP-Cre (see above) mice to prevent Na_v_1.7 expression in OSNs (mice were kindly provided by Drs. Frank Zufall and Trese Leinders-Zufall).

All experiments were in accordance with the Swiss Animal Protection Ordinance and were approved by the University of Geneva and Geneva state ethics committees.

### Recordings in Awake Mice

During the 3 days prior to the recordings, mice were habituated to be head restrained on the recording setup for 2× 30 min per day (Gschwend et al., 2012). The day of the recording, mice were briefly anesthetized with isoflurane (3% to 4% induction; 1% to 2% maintenance). The bone was thinned with a scalpel blade to achieve good optical access. Animals were left to fully recover for at least 30 min before starting imaging sessions. See also Supplemental Experimental Procedures.

### Recordings in Anesthetized Mice

Animals were deeply anesthetized, and a circular craniotomy (using a 2-mm biopsy punch; Harris UNI-CORE) was made over the OB, leaving the dura intact. The craniotomy was filled with ACSF and covered with a glass coverslip (5 mm of diameter). See also Supplemental Experimental Procedures.

All the drugs used, except for CD^2+^ and Ba^2+^ (purchased from Sigma Aldrich) were purchased from Tocris Bioscience. Drugs were dissolved in ACSF containing in mM 125 NaCl, 10 glucose, 10 HEPES, 5 KCl, 2 CaCl_2_, and 2 MgCl_2_. Ten microliters of ACSF with or without drugs were topically applied to the surface of one OB. After removing the cover glass, the dura mater was dried by gently puffing air. Drugs were applied on the dried dura, and a cover glass was placed back on the craniotomy. This procedure allows the dura membrane to be more permeable to pharmacological agents.

For water intoxication experiments, a volume of 20% of mouse body weight of distilled water (dH_2_O) or sterile isotonic solution (308 mosm/l, 0.9% NaCl; Sintetica-Bioren SA) was injected i.p. (Manley et al., 2000). The pH (7.3) and temperature (37° C) of the solution were checked before each injection.

### Imaging Experiments

To perform intrinsic signal imaging, the OB was illuminated with incident light using a stable 100-W halogen lamp with light guides (Bathellier et al., 2008). The wavelength of incident light was varied depending on experimental needs using the following interference filters: 546 (BP 30 nm); 605 (BP 30 nm); 700 (BP 30 nm); and 810 nm (BP 30 nm). Images were acquired at 5 Hz for 10 s using the Imager 3001F system (Optical Imaging) or at 14 Hz using the Micam Ultima system (Brainvision) mounted both on a custom-built macroscope (Patterson et al., 2013; Navitar 17 mm or 25 mm, bottom lenses; Nikon 135 mm, upper lens; total magnification 7.9x or 5.4x). See also Supplemental Experimental Procedures.

### Odor Delivery

All monomolecular odorants used in the experiments (amyl acetate, ethyl butyrate, isoamyl acetate, carvone-, 3-hexanone, acetophenone, and methyl benzoate) were from Sigma-Aldrich. See also Supplemental Experimental Procedures.

### Data Analysis and Statistical Analysis

All analyses were performed with custom Matlab (MathWorks) scripts and Statistica. See also Supplemental Experimental Procedures.

## Supplemental Information

Supplemental Information includes Supplemental Experimental Procedures and five figures and can be found with this article online at http://dx.doi.org/10.1016/j.celrep.2015.06.016.

Supplemental Information

## Highlights

- We studied the origin of intrinsic optical signals (IOSs) in the mouse olfactory bulb
- IOSs are independent of neurovascular coupling and astrocyte function
- IOSs are independent of neurotransmitter release and post-synaptic neuronal activity
- IOSs arise from activity-dependent swelling of sensory neuron axons
