# Supplementary material for: Sensory-Evoked Intrinsic Imaging Signals in the Olfactory Bulb Are Independent of Neurovascular Coupling
Source: Cell Rep. Author manuscript; Available in PMC 2016 Oct 17. (PMC5066842; doi:10.1016/j.celrep.2015.06.016)
Supplement: Supplemental Information [file NIHMS69936-supplement-Supplemental_Information.pdf]

Cell Reports

Supplemental Information

Sensory-Evoked Intrinsic Imaging Signals in the Olfactory Bulb Are Independent of Neurovascular Coupling

Roberto Vincis, Samuel Lagier, Dimitri Van De Ville, Ivan Rodriguez, and Alan Carleton

Supplemental information

Sensory-evoked intrinsic imaging signals in the olfactory bulb are independent of neurovascular coupling

Roberto Vincis, Samuel Lagier, Dimitri Van de Ville, Ivan Rodriguez and Alan Carleton

Contents:

Supplemental Experimental procedures

Supplemental References

Supplemental Figures

Supplemental Experimental procedures

Recordings in awake mice

Animals were anesthetized by intraperitoneal injection (i.p.) of 3.1µl/g body weight of a mixture (sleep mix) consisting of 60µl medetomidin (Dormitor®, Pfizer AG, Zurich, Switzerland; 1mg/ml), 160µl midazolam (Dormicum®, Roche Pharma AG, Switzerland; 5mg/ml) and 40µl fentanyl (Sintenyl®, Sintetica S.A., Mendrisio, Switzerland; 50µg/ml). 300µl of carprofen (Rimadyl®, Pfizer, Switzerland; 0.5mg/ml) was injected i.p. to prevent inflammatory processes. The skin overlaying the skull was removed under local anaesthesia using carbostesin (AstraZeneca, Zug, Switzerland). A steel head-post was then fixed on the bone by embedding its base in dental cement (Omni-Etch Dentin, OmniDent). The rest of the skull was also covered with dental cement except the part overlaying the OB. Animals were woken up by i.p. injection of 10µl/g body weight of a mixture consisting of 400µL flumazenilum (Anexate®, Roche Pharma AG, Switzerland; 0.1mg/ml), 10µL atipamezole (Alzane®, Graeub, Switzerland ; 5mg/mL) and 3µL naloxone (OrPha Swiss GmgH, Switzerland ; 0.4mg/mL) then put back in their cage and allowed to recover for couple of days.

Recordings in anesthetized mice

Animals were deeply anesthetized by intraperitoneal injection (i.p.) of 3.1µl/g body weight of sleep mix (see above). A local anesthetic, carbostesin (AstraZeneca, Zug, Switzerland), was subcutaneously injected before any skin incision. Anesthesia was maintained by periodic dosage (~30µl i.p. every 30 minutes) of mixture containing only Midazolam (5mg/ml) and Medetomidin (1mg/ml). A circular craniotomy (using a 2mm biopsy punch, Harris UNI-CORE™) was made over the OB, leaving the dura intact. The craniotomy was filled with ACSF and covered with a glass cover slip (5mm of diameter). Body temperature was maintained at 36° with a heating blanket throughout the experiment. Breathing rate (all experiments) and heartbeat rate (water intoxication experiment) were carefully monitored throughout all experimental sessions.

The concentration of drugs used are the following: 2,3-dioxo-6-nitro-1,2,3,4-tetrahydrobenzoquinoxaline-7-sulfonamide disodium salt (NBQX, 100µM), dl-2-amino-5-phosphonovaleric acid (dl-APV, 1mM), (3-Aminopropyl)(diethoxymethyl)phosphinic acid (CGP 35348, 1mM), (*R*)-5,6,6a,7-Tetrahydro-6-methyl-4*H*-dibenzo [*de,g*] quinoline-10,11-diol hydrochloride (apomorphine, 30µM), (*RS*)-4-Amino-3-(4-chlorophenyl) butanoic acid (baclofen, 10µM),(*S*)-(-)-5-Aminosulfonyl-*N*-[(1-ethyl-2-pyrrolidinyl)methyl]-2-methoxybenzamide (sulpiride, 1mM), (*RS*)-α-Methyl-4-carboxyphenylglycine- disodium salt (MCPG, 200µM), (*RS*)-α-Cyclopropyl-4-phosphonophenyl-glycine (CPPG, 10µM), (2*S*)-2-Amino-2-[(1*S*,2*S*)-2-carboxycy-cloprop-1-yl]-3-(xanth-9-yl) propanoic acid disodium salt (LY 341495, 2 µM), Cadmium chloride hemi (penta-hydrate) (Cd^{2+}, 2mM), (2*S*,3*S*,4*R*)-2-Carboxy-4-isopropyl-3--pyrrolidineacetic acid (DHK, 1mM), 8,8'-[Carbonyl*bis*[imino-3,1-phenylen-ecarbonylimino(4-methyl-3,1-phenylene)carbonylimin-o]]*bis*-1,3,5- naphthalenetrisulfonic acid hexasodium salt (Suramin, 2mM), Barium chloride dehydrate (Ba^{2+}, 1mM), DL-*threo*-β-Benzyloxyaspartic acid (TBOA, 5mM), 9-Chloro-2-(2-furanyl)-[1,2,4]triazolo[1,5-*c*]quinazolin-5-amine (CGS, 0.5mM), Octahydro-12-(hydroxymethyl)-2-imin-o-5,9:7,10a-dimethano-10a*H*-[1,3]dioxocino[6,5-*d*]py-rimidine-4,7,10,11,12-pentol (TTX, 1µM).

Imaging experiments

The blood vessel pattern was recorded under green light (546nm interference filter) at the beginning of each experimental trial session and used to realign images. The final dimension of the collected pixel matrix was 256×256 pixels for the Imager 3001F system and 100×100 for the Micam System. Pixel values of collected images were computed as ΔR/R for IOS. Considering t_{ON} and t_{OFF} as odor onset and odor offset respectively, ΔR/R was computed as follows: $(R_1-R_0)/R_0$, where R_0 is the average pixel reflectance value before odor stimulation [from $(0.2 * t_{ON})$ to $(0.8 * t_{ON})$], and R_1 is the average pixel reflectance value around the maximum response [from $(t_{OFF} - 0.1 * (t_{OFF}-t_{ON}))$ to $(t_{OFF} + 0.7 * (t_{OFF}-t_{ON}))$]. Epifluorescence imaging (SpH and GcaMP3) was done only with the Micam Ultima system. Here the OB was imaged using a 480nm (BP 40nm) excitation filter, a dichroic mirror (Q 505 LP) and a 535nm (BP 50nm) emission filter. The excitation light intensity was adjusted to have in each experimental session, an average resting fluorescence lower than 20% of the sensitivity of our camera. No significant bleaching was observed with these settings. Images were acquired at 14Hz (OMP-SpH and GFAP-GCaMP3) and at 33Hz (OMP-

GCaMP3 and PCDH21-GCaMP3). Pixel values of collected images are represented as ΔF/F. ΔF/F for OMP-SpH and GFAP-GCaMP3 is computed in the same way as ΔR/R for IOS data. F_1 for OMP-GCaMP3 is computed by averaging from (t_{ON}+ 0.3 * (t_{OFF}-t_{ON})) to t_{OFF}.

Odor delivery

All monomolecular odorants used in the experiments (amyl acetate, ethyl butyrate, isoamyl acetate, carvone-, 3-hexanone, acetophenone and methyl benzoate) were from Sigma Aldrich (Germany). Odorants were presented using computer-controlled custom-made olfactometers (Bathellier et al., 2007; Tatti et al., 2014). Odorants were diluted in clean dry air (20-40x) and presented for 5s. Breathing was recorded via a directional airflow sensor (Gschwend et al., 2012; Tatti et al., 2014) (Honeywell; AWM2100V). Heartbeat was recorded with an ECG amplifier connected to 2 subcutaneous AgCl electrodes, one over the left shoulder and one over the right hip (Sigmann elektronik, Germany). For *in vivo* electric stimulation of OSN axons, 12 trials of a single current pulse (100ms duration, 5-50V intensity, 15s inter trial interval) were delivered to the OB dorsal surface using a bipolar tungsten electrode. The electrode was placed on the anterior-lateral portion of the bulb, where olfactory nerve bundles innervating the dorsal surface of the OB are located.

Data analysis and statistical analysis

All analyses were performed using custom Matlab (MathWorks, Inc., Natick, MA) scripts. All images in figures 1-5 were spatially filtered with a band-pass filter (between 3.7 and 370µm for IOS and between 12.5 and 1250µm for fluorescence). For epifluorescence data, the entire medial half and the activated ROIs on the lateral side were excluded from the high-pass filter. We also looked at the raw and diffuse component of IOS (Figures S2, S5 and S7). The raw IOS is simply unfiltered signal (ΔR/R is computed on raw images) and the diffuse component is obtained with a band-pass filter between 100µm and the smaller dimension of the images (i.e. 1250µm for fluorescence and ~570µm for IOS). A simple exponential fit (y = a*exp (-t/b) + c) was applied on IOS data from figure 1. The latency was calculated as -b*log (-c/a). We confirmed the latency difference between glomeruli and blood vessel using a thresholding method (threshold set at 3*SD of the baseline, data not shown). This latter approach is non-biased but does not report absolute latencies. Data in figures 3-6 are normalized relative to the baseline, prior to pharmacological application or relative to a previous pharmacological condition. Shapiro-Wilk test was used to assess normality of the data. For all parametric tests, homogeneity of variance was tested using *F*-test or a test of sphericity (for one-way repeated measures ANOVA). In case of equal variance, a *t*-test was used. Otherwise, a Mann-Whitney *U* test was used for unpaired comparison. For paired comparisons (large samples), a Wilcoxon signed-rank test was used. A one-way repeated measure ANOVA was used for figure 2F (green bars) with least significant difference (LSD) post-hoc analysis. Data are represented as mean ± SEM (standard error of the mean), with n representing either the number of glomeruli or the number of olfactory bulbs (see figure legends for details).
Independent Component Analysis (ICA) is a multivariate data-driven exploratory technique that allows separating the data into components based a surrogate criterion for statistical independence. The data is represented as a linear combination of components where each of them consists of a spatial map (source) and an associated time course (mixing coefficients). We applied the FastICA algorithm (Hyvarinen, 1999) including dimensionality reduction using principal components analysis to every preprocessed dataset. We found that 98.5% of the variance could be explained by a small number of components; i.e., 4.6 components were sufficient on average. Finally, the components' spatial maps were Z-scored (threshold at |Z|>3) and visually assessed in terms of the spatial origin of signal contributions.

Supplemental References

Bathellier, B., Van De Ville, D., Blu, T., Unser, M., and Carleton, A. (2007). Wavelet-based multi-resolution statistics for optical imaging signals: Application to automated detection of odour activated glomeruli in the mouse olfactory bulb. Neuroimage *34*, 1020–1035.

Hyvarinen, A. (1999). Fast and Robust Fixed-Point Algorithms for Independent Component Analysis. *10*, 626–634.

Gschwend, O., Beroud, J., and Carleton, A. (2012). Encoding odorant identity by spiking packets of rate-invariant neurons in awake mice. PLoS One *7*, e30155.

Tatti, R., Bhaukaurally, K., Gschwend, O., Seal, R.P., Edwards, R.H., Rodriguez, I., and Carleton, A. (2014). A population of glomerular glutamatergic neurons controls sensory information transfer in the mouse olfactory bulb. Nature communications *5*, 3791.

Supplemental Figures

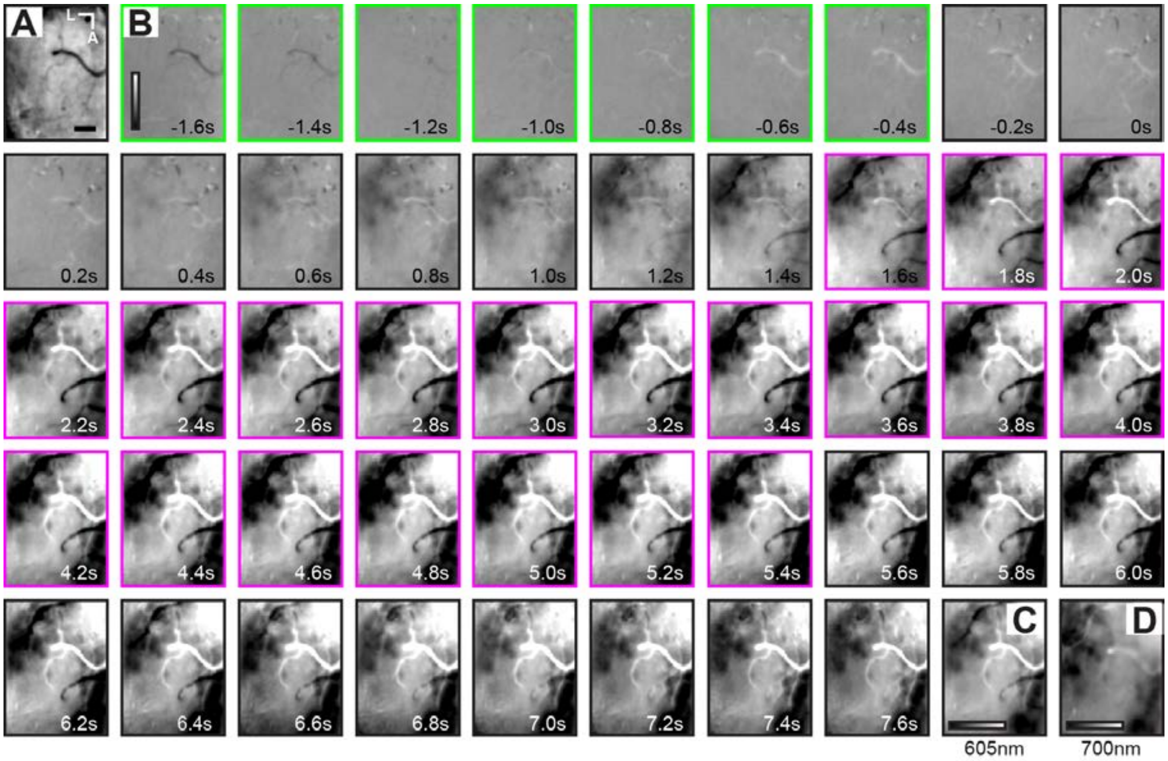

Figure S1. Evolution of odor-evoked parenchymal IOS over time in an awake mouse, Related to Figure 1

(A) Dorsal OB blood vessel pattern. Scale bar : 200 µM.

(B) Successive frames of glomerular activity evoked by ethyl butyrate (5% in air) computed as ΔR/R ($(R-R_0)/R_0$). The wavelength of the incident light is 605nm (same data as in Figure 1A). Times are shown relative to odor onset. Images framed in green and in magenta cover the time ranges used for R_0 and R_1 computation respectively (see Experimental procedures). LUT : -0.005 to 0.01.

(C) Average of the magenta-framed images shown in B. The same time range (1.6 to 5.4 s, relative to odor onset) is used to produce the average map images shown in figures 1-5; LUT : -0.0055 to 0.0035.

(D) Average ΔR/R measured at 700nm, shown for comparison. LUT : -0.004 to 0.0035.

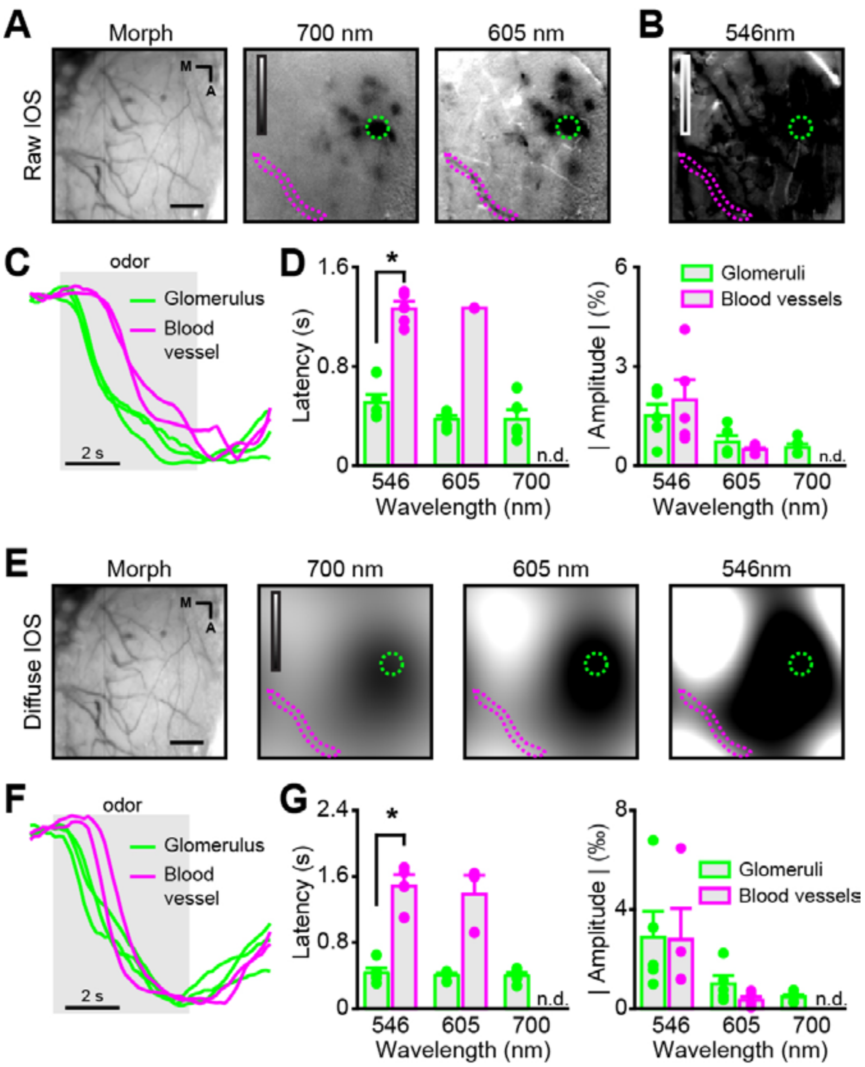

Figure S2. Parenchymal IOS dynamics do not depend on image processing, Related to Figure 2

(A) *Left* (Morph): Blood vessel pattern of the dorsal OB. *Right*: unfiltered ("raw") IOS glomerular map evoked by methyl benzoate (5% in air) recorded at 2 different wavelengths (700 and 605nm) in the same animal (same data as in Fig. 2*A*). LUT: -0.0035 to 0.0015 ΔR/R. Green and magenta dotted lines are the same ROIs as in Figure 2A.

(B) IOS map from the same animal as in A, imaged at 546nm. LUT: -0.01 to 0.06 ΔR/R.

(*C*) Raw IOS time course of the ROIs marked in A and B, at the different wavelengths, normalized for comparison – 546, 605 and 700nm for the glomerulus (*green*) and 546 and 605nm for the blood vessel (*magenta*). Note the delay from odor onset of the blood vessel traces (*magenta*) compared to glomerular ones (*green*).

(D) *Left*: Average response latencies of raw IOS computed for glomeruli (*green*) and blood vessels (*magenta*) (Unpaired *t* test, glomeruli *vs*. blood vessels at 546nm, n = 5 mice, t(8) = -8.5, p = 2.8e-5). *Right*: Average response absolute amplitudes of raw IOS (unpaired *t* test, glomeruli *vs*. blood vessels, 546nm, n = 5 mice, t(8) = 0.44, p = -0.79; unpaired *t* test, glomeruli *vs.* blood vessels, 605nm, n = 5 mice, t(8) = 1.13, p = 0.28).

(E) *Left* (Morph): Same blood vessel pattern as in *A*. *Right*: low-frequency band pass filtered (*i.e.* "diffuse"; filtered between 100µm and the width of the image) IOS glomerular maps evoked by methyl benzoate (5% in air) recorded at 3 different wavelengths (700, 605, 546nm) in the same animal, displayed in A. LUT: -0.0005 to 0.0005 ΔR/R. Green and magenta dotted lines are the same ROIs as in A.

(F) Diffuse IOS time course of the ROIs marked in E, at the different wavelengths, normalized for comparison – 546, 605 and 700nm for the glomerulus (*green*) and 546 and 605nm for the blood vessel (*magenta*). Note the delay from odor onset of the blood vessel traces (*magenta*) compared to glomerular ones (*green*).

(G) *Left*: Average response latencies of diffuse IOS computed for glomeruli (*green*) and blood vessels (*magenta*) (unpaired *t* test, glomeruli *vs* blood vessels, 546nm, n = 5 and 4 mice for glomeruli and blood vessels respectively, t(7) = -7.69, p = 1.2e-4 ; Mann-Whitney *U* test, U(8) = 10, p = 0.67). *Right*: Average response absolute amplitudes of diffuse IOS (unpaired *t* test, glomeruli *vs.* blood vessels, n = 5 mice except for blood vessels at 546nm where n = 4 mice, t(7) = -0.058, p = 0.96 and t(8) = 2.07, p = 0.07 at 546 and 605nm, respectively).

Scale bars (A, E): 100µm. The light gray box (C, F) represents odor presentation. Data are presented as mean ± SEM.

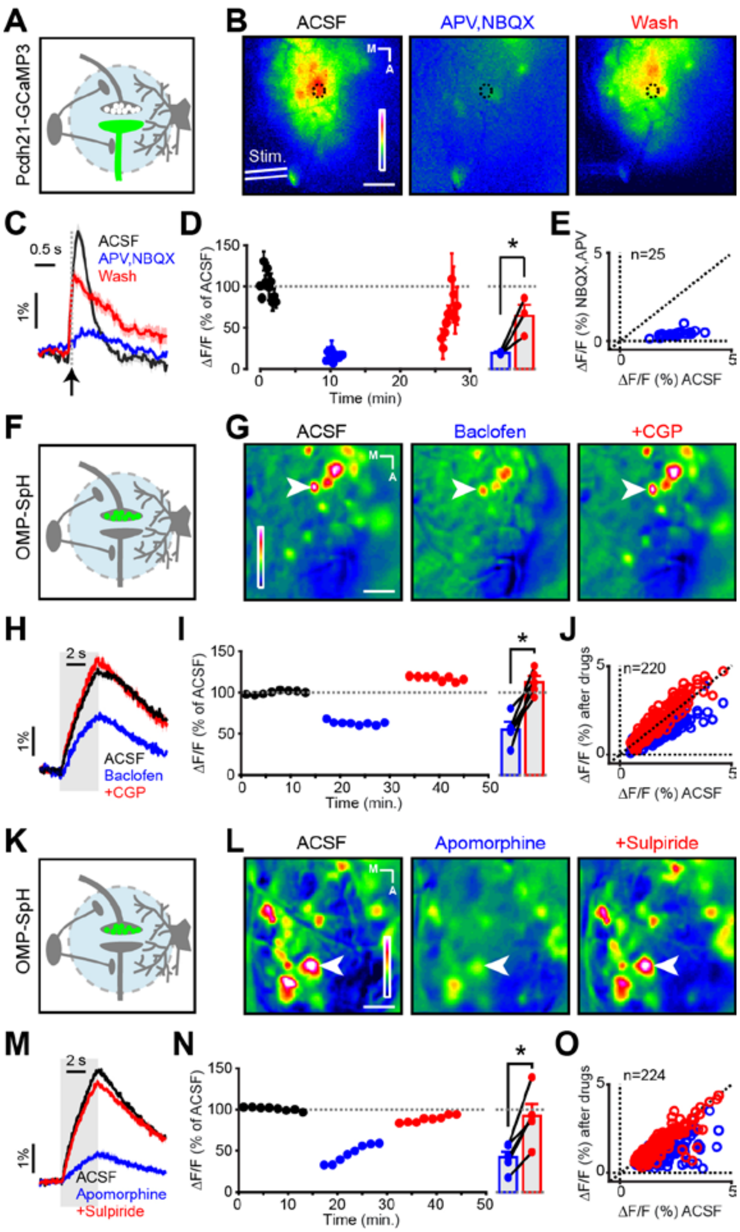

Figure S3. Experimental validation of *in vivo* pharmacological approach, Related to Figure 3

(A) schematic representation of the OB glomerular circuitry. The location of the fluorescent reporter is represented in green for the different mouse lines.

(B) Average map of odor-evoked activity reported by PCDH21-GCaMP3 in control condition (ACSF, *left*), in presence of APV (1mM) and NBQX (0.1mM, *center*), and after wash out (Wash, *right*). Black circle points to an example of stimulus-evoked activated ROI. LUT: -0.01 to 0.05 ΔF/F. The location of the stimulating electrodes (Stim) is highlighted in B.

(C) Time courses of stimulus-evoked activity from the ROI marked in B, for 3 conditions (ACSF, *black*; APV and NBQX, *blue*; and Wash, *red*; mean ± SEM of 10 trials). Light gray vertical dotted line and black arrow mark the onset of electrical stimulation.

(D) Single trials (*left*) and average (*right*; bar graph) across mice values of ROI amplitude responses (ΔF/F) during the 3 conditions shown in C (n = 3 bulbs from 3 mice, paired *t* test, t(2) = 3.5, p = 0.074).

(E) Average OMP-SpH response amplitudes in presence of APV and NBQX (blue circles) plotted against amplitude values in control condition (ACSF) (n = 25 glomeruli from 3 mice, Wilcoxon signed-rank test, Z(25)=-4.8, p =1.2e-6).

(F) Same as in A.

(G) Average map of OMP-SpH odor-evoked activity in control condition (ACSF, *left*), in presence of baclofen (0.1mM; *center*), and in presence of CGP (1mM; *right*). White arrows point to example of odorant-evoked glomeruli. LUT: -0.01 to 0.03 ΔF/F.

(H) Time course of odor-evoked activity from the glomerulus marked in G, for 3 conditions (ACSF, *black*; baclofen, *blue* and CGP, *red*; mean ± SEM of 8 trials).

(I) Glomeruli response amplitudes presented as single trials (*left*) and average across mice (*right*; bar graph; n = 5 bulbs from 3 mice, paired *t* test, t(4) = -5.0, p = 0.074) for the 3 conditions shown in H.

(J) Average amplitude in presence of baclofen (blue circles) and CGP (red circles), plotted against amplitude in control condition (ACSF) (n = 220 glomeruli from 3mice, Wilcoxon signed-rank test, Z(220)=-4.4, p = 6.4e-34).

(K) Same as in A.

(L) Average map of OMP-SpH odor-evoked activity in control condition (ACSF, *left*), in presence of apomorphine (0.1mM; *center*), and in presence of sulpiride (1mM; *right*). White arrow points to an example of odorant-evoked glomeruli. LUT: -0.01 to 0.03 ΔF/F.

(M) Time course of odor-evoked activity from the glomerulus marked in L, for 3 conditions (ACSF, *black*; apomorphine, *blue* and sulpiride, *red*; mean ± SEM of 8 trials).

(N) Glomeruli response amplitudes presented as single trials (*left*) and average across mice (*right*; bar graph; n = 5 bulbs from 3 mice, paired *t* test, t(4) = -5.5, p = 0.0052) for the 3 conditions shown in H.

(O) Average response amplitudes in presence of apomorphine (*blue*) and sulpiride (*red*), plotted against amplitudes in control condition (ACSF) (n = 224 glomeruli from 3 mice, Wilcoxon signed-rank test, Z(224)=-12.1, p =1.8e-33). Scale bar: 100µm. Light gray box represents odor presentation.

Data are presented as mean ± SEM.

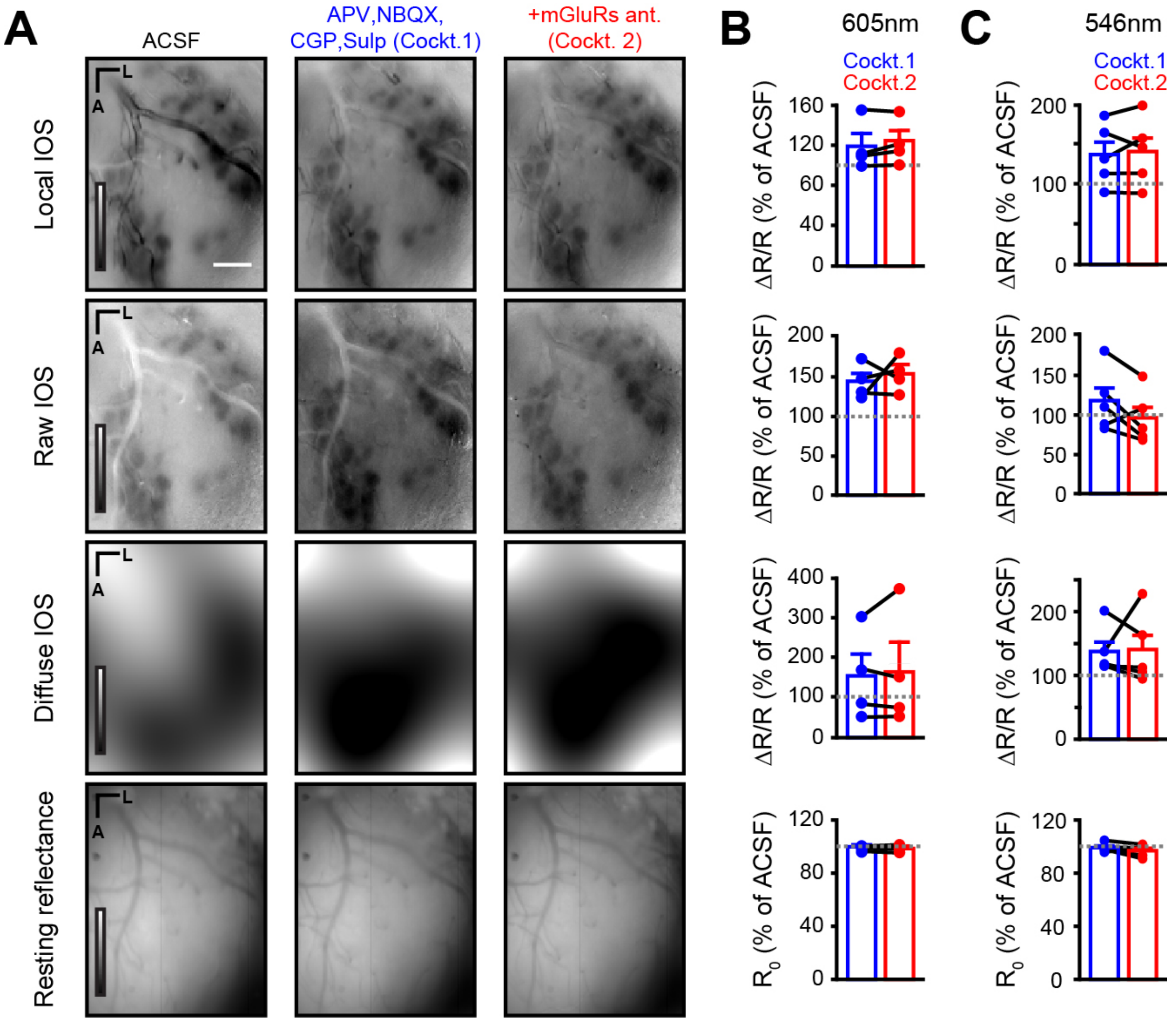

Figure S4. IOS pharmacological dissection does not depend on the incident light wavelength, Related to Figure 3

(A) Average map of odor-evoked IOS imaged at 605nm with a high-frequency band-pass filter (*local IOS*, 1st row), with no filter (*raw IOS*, 2d row), with a low-frequency band-pass filter (*diffuse IOS*, 3rd row) and average resting fluorescence (also referred to as R_0, bottom row) under 3 successive pharmacological conditions: ACSF (first column), Cockt.1 (middle column) and (Cockt.2, right column). Scale bar: 100µm. LUT (top to bottom): -9e-3 to 9e-3, -0.02 to 0.001, -2e-3 to 2e-3 ΔR/R and 5e+4 to 21e+4.

(B) Average values of glomeruli amplitude response imaged at 605nm after application of Cockt.1 (*blue*) and Cockt.2 (*red*) across mice for different signal processing (*local*, *raw* and *diffuse IOS*) and resting reflectance. Values are normalized relative to control condition (ACSF). (Paired *t* test, Cockt.1 *vs.* Cockt.2, n = 4 bulbs from 3 mice, t(3) = -1.48, p = 0.23; t(3) = -0.54, p = 0.62; t(3) = -0.52, p = 0.61; t(3) = 0.85, p = 0.45 for local, raw, diffuse IOS and R_0 respectively.

(C) Average values of glomeruli amplitude response imaged at 546nm after application of Cockt.1 (blue) and Cockt.2 (red) across mice for different signal processing (*local*, *raw* and *diffuse IOS*) and resting reflectance. Values are normalized relative to control condition (ACSF). (Paired *t* test, Cockt.1 *vs.* Cockt.2, n = 5 mice, t(4) = -0.51, p = 0.64; t(4) = 1.88, p = 0.13; t(4) = -0.14, p = 0.9; t(4) = 1.2, p = 0.3 for local, raw, diffuse IOS and R_0 respectively.

Data are presented as mean ± SEM.

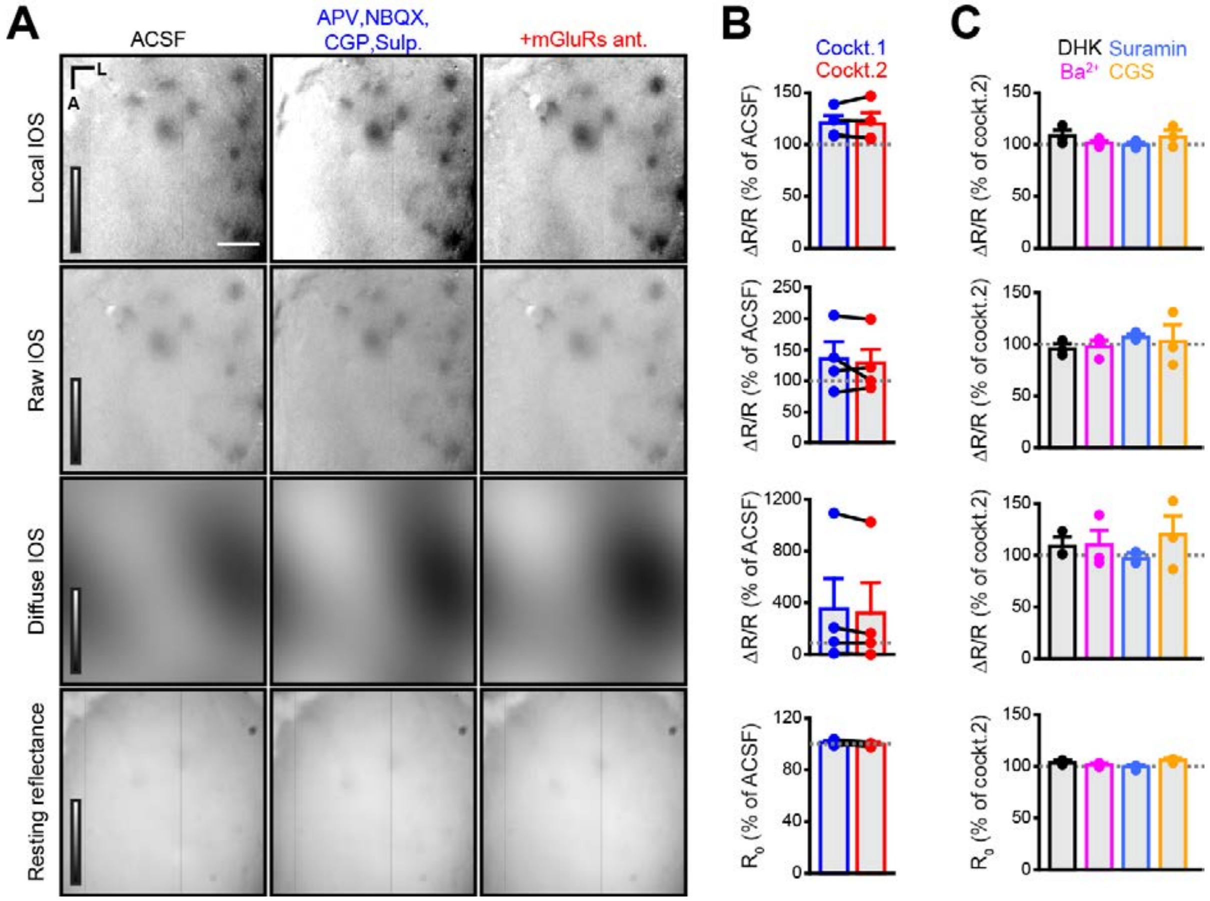

Figure S5. IOS pharmacological dissection does not depend on image processing, Related to Figures 3 and 4

(A) Average map of odor-evoked IOS with a high-frequency band-pass filter (*local IOS*, first row), with no filter (*raw IOS*, 2^{d} row), with a low-frequency band-pass filter (*diffuse IOS*, 3rd row) and average resting fluorescence (also referred to as R_0, bottom row) under 3 successive pharmacological conditions: ACSF (first column), APV, NBQX, CGP and sulpiride (1, 0.1, 1 and 1mM respectively, also referred to as Cockt.1, middle column) and the addition of LY 341495, MCPG and CPPG (2, 200 and 10µM respectively) to the previous antagonists (+*mGluRs ant.*, also referred to as Cockt.2, right column). The experiment displayed here is the same as in Figure 3A. Scale bar: 100µm. LUT (top to bottom): -0.01 to 0.006, -0.009 to 0.002, -7e-4 to 7e-4 ΔR/R and 15e+4 to 35e+4.

(B) Average values of glomeruli amplitude response after application of Cockt.1 (blue) and Cockt.2 (red) across mice for different signal processing (*local*, *raw* and *diffuse IOS*) and resting reflectance. Values are normalized relative to control condition (ACSF) (Paired *t* test, Cockt.1 *vs.* Cockt.2, n = 4 bulbs from 3 mice, t(3) = -0.03, p = 0.97; t(3) = 0.76, p = 0.49; t(3) = 1.93, p = 0.14; t(3) = 2.08, p = 0.12 for local, raw, diffuse IOS and R_0 respectively.

(C) Average values of glomeruli amplitude response after application of Cockt.2 and DHK (1mM, black), Cockt.2 and Ba^{2+} (1mM, magenta), Cockt.2 and suramin (2mM, cyan) and Cockt.2 and CGS (0.5mM, dark yellow). Data are normalized to the Cockt.2 condition and are presented, from top to bottom, as local, raw and diffuse IOS and resting fluorescence. The data used here are the same as for Figure 4 H, 4L, 4P and 4T.

Data are presented as mean ± SEM.
